# Author Correction: Massively parallel interrogation of protein fragment secretability using SECRiFY reveals features influencing secretory system transit

**DOI:** 10.1038/s41467-023-36844-y

**Published:** 2023-02-24

**Authors:** Morgane Boone, Pathmanaban Ramasamy, Jasper Zuallaert, Robbin Bouwmeester, Berre Van Moer, Davy Maddelein, Demet Turan, Niels Hulstaert, Hannah Eeckhaut, Elien Vandermarliere, Lennart Martens, Sven Degroeve, Wesley De Neve, Wim Vranken, Nico Callewaert

**Affiliations:** 1 Center for Medical Biotechnology, VIB, Zwijnaarde, Belgium; 2 Department of Biochemistry and Microbiology, Faculty of Sciences, Ghent University, Ghent, Belgium; 3 Department of Biomolecular Medicine, Faculty of Medicine and Health Sciences, Ghent University, Ghent, Belgium; 4 Structural Biology Brussels, VUB, Brussels, Belgium; 5 Structural Biology Research Center, VIB, Brussels, Belgium; 6 Interuniversity Institute of Bioinformatics in Brussels (IB)2, ULB-VUB, Brussels, Belgium; 7 Center for Biotech Data Science, Ghent University Global Campus, Songdo, Incheon, South Korea; 8 IDLab, ELIS, UGent, Ghent, Belgium; 9 Present Address: Department of Biochemistry and Biophysics, UCSF, San Francisco, CA USA

**Keywords:** High-throughput screening, Expression systems, Functional genomics, Machine learning, Proteomics

Correction to: *Nature Communications* 10.1038/s41467-021-26720-y, published online 05 November 2021

In this article the author name Wesley De Neve was incorrectly written as Wim De Neve. The original article has been corrected.

