# Supplementary material for: Massively parallel interrogation of protein fragment secretability using SECRiFY reveals features influencing secretory system transit
Source: Nat Commun. 2021 Nov 5;12:6414. doi: 10.1038/s41467-021-26720-y (PMC8571348; doi:10.1038/s41467-021-26720-y)
Supplement: Supplementary file 1 — Supplementary Information [file 41467_2021_26720_MOESM1_ESM.pdf]

Supplementary Information for

Massively parallel interrogation of protein fragment secretability using SECRiFY reveals features influencing secretory system transit

Boone, M.[1,2,7,*]; Ramasamy, P.[1,3,4,5,6], Zuallaert, J.[1,2,8,9], Bouwmeester, R.[1,3], Van Moer, B.[1,2], Maddelein, D.[1,3], Turan, D.[1,3], Hulstaert, N.[1,3], Eeckhaut, H.[1,2], Vandermarliere, E.[1,3], Martens, L.[1,3], Degroeve, S.[1,3], De Neve, W.[8,9], Vranken, W.[4,5,6], Callewaert, N.[1,2,*]

1. Center for Medical Biotechnology, VIB, Zwijnaarde, Belgium

2. Department of Biochemistry and Microbiology, Faculty of Sciences, Ghent University, Ghent, Belgium

3. Department of Biomolecular Medicine, Faculty of Medicine and Health Sciences, Ghent University, Ghent, Belgium

4. Structural Biology Brussels, VUB, Brussels, Belgium

5. Structural Biology Research Center, VIB, Brussels, Belgium

6. Interuniversity Institute of Bioinformatics in Brussels (IB)2, ULB-VUB, Brussels, Belgium

7. Present address: Department of Biochemistry and Biophysics, UCSF, San Francisco, CA, USA

8. Center for Biotech Data Science, Ghent University Global Campus, Songdo, Incheon, South Korea

9. IDLab, ELIS, UGent, Ghent, Belgium

* corresponding authors: morganeboone@gmail.com; nico.callewaert@vib-ugent.be

This pdf file includes

Suppl. Figs 1-18

Suppl. Tables 1-20

Suppl. References

Supplementary Figures

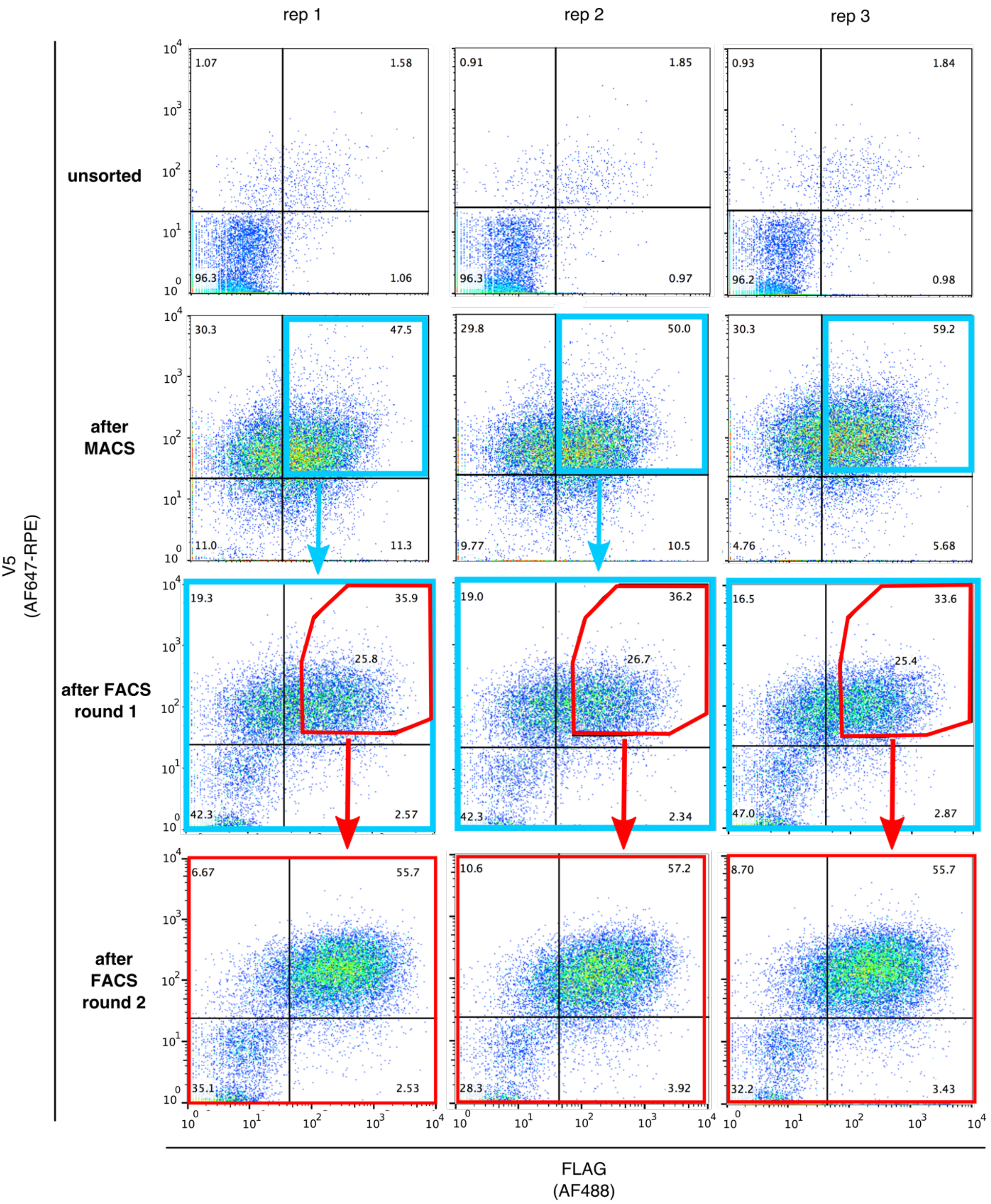

Supplementary Fig. 1. Sorting of fragment-displaying *S. cerevisiae* library clones. The *S. cerevisiae* cDNA fragment yeast library was sorted for FLAG$^+$V5$^+$ cells in three replicate experiments (rep 1, rep2 and rep 3). In the first round, V5$^+$ cells were enriched by MACS (row 2, ‘after MACS’), and further directly sorted out for FLAG$^+$V5$^+$ cells by FACS. Sorted cells were frozen after recovery, regrown for induction and staining, and subjected to another round of FACS with a

slightly more stringent (red) gate (row 3, ‘after FACS round 1’). These round 2 sorted cells were frozen for storage, again regrown for induction and staining, and examined for purity by flow cytometry (row 4, ‘after FACS round 2).

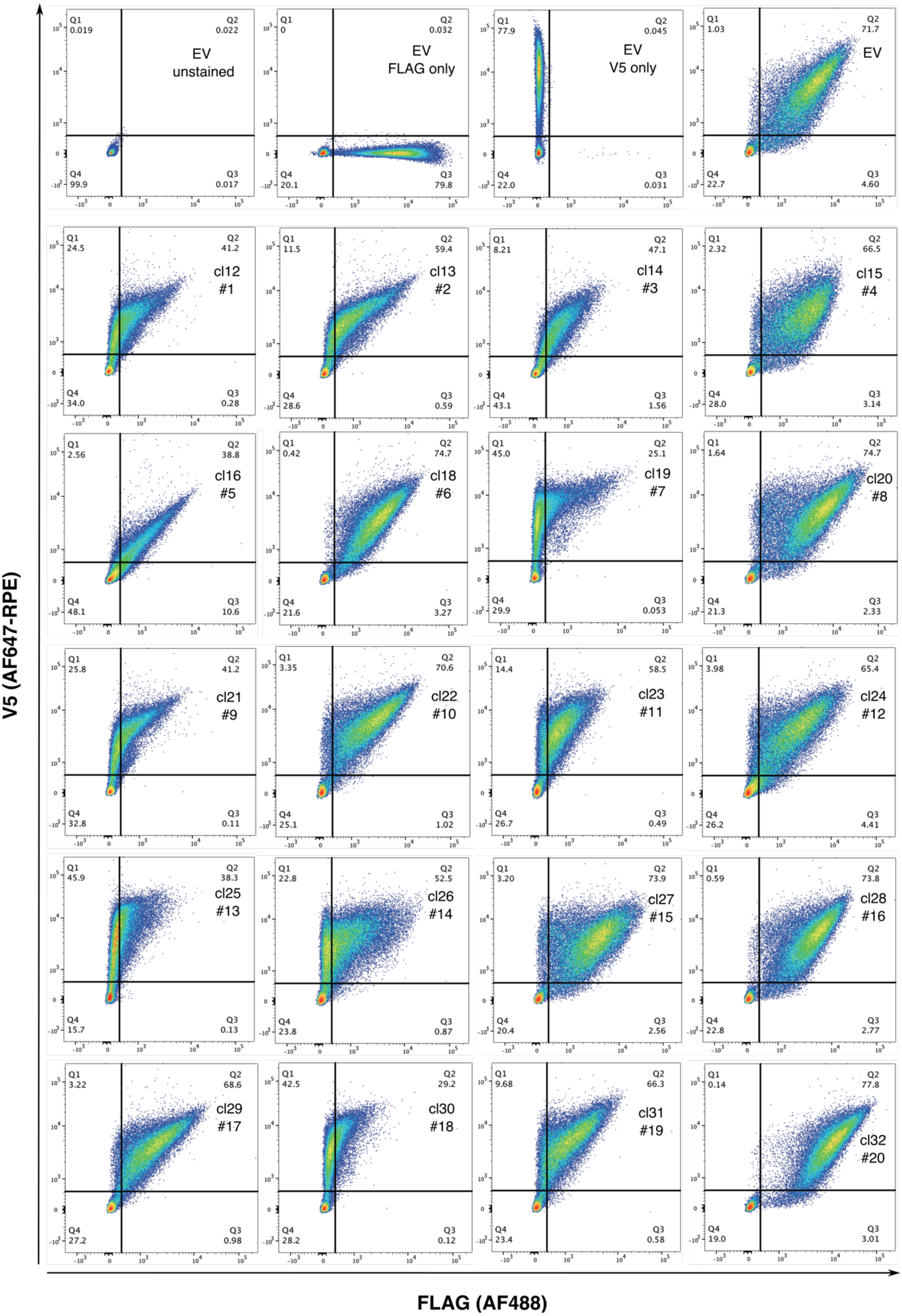
V5 (AF647-RPE)
FLAG (AF488)
EV unstained
EV FLAG only
EV V5 only
EV
cl12 #1
cl13 #2
cl14 #3
cl15 #4
cl16 #5
cl18 #6
cl19 #7
cl20 #8
cl21 #9
cl22 #10
cl23 #11
cl24 #12
cl25 #13
cl26 #14
cl27 #15
cl28 #16
cl29 #17
cl30 #18
cl31 #19
cl32 #20

Supplementary Fig. 2. Flow cytometry plots of select randomly picked clones after two rounds of sorting. Top row: FLAG-V5-Sag1 control strain (EV= empty vector) unstained, single stained, and double stained controls. Other rows are the double-stained single clones. Clone number (cl...) matches the clone identity in **Suppl. Table 4**, and the number below (#...) indicates the fragment as used for secretion check on western blot (**Fig. 1d** and **Suppl. Table 5**). Clone 17 was omitted as it was not detected in the sequencing data.

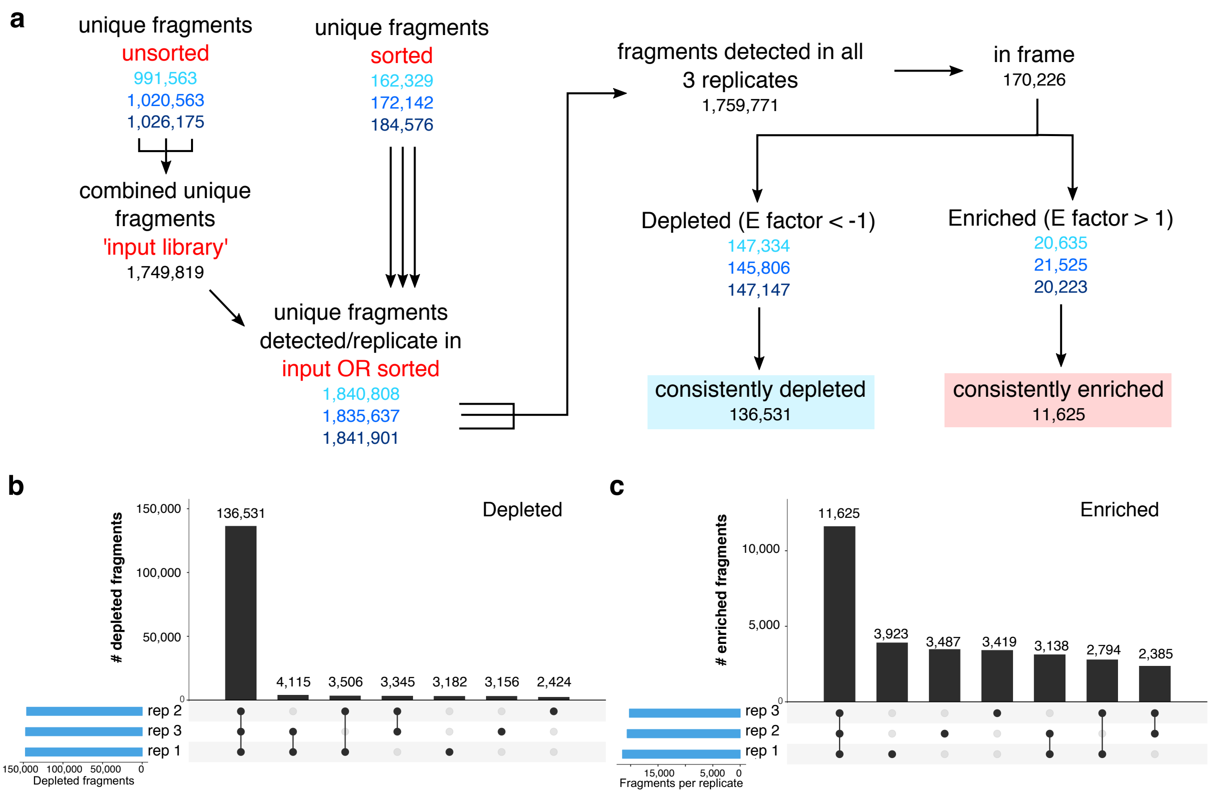

Supplementary Fig. 3. Enriched/depleted fragment classification and replicate statistics in *S. cerevisiae* screens. a) Fragment processing workflow for classification into enriched/depleted fragments. The number of fragments in each of the three replicate screens is shown in different shades of blue. A fragment is considered detected in a replicate when it is detected in either the sorted sample of that experiment, and/or in the input library. Three independent samples of this input library were sequenced, and we combined all of the fragments detected in the total sequence read pool to enhance the sequencing depth of this highly diverse unsorted input fragment library. Consistently depleted (or enriched) fragments are in-frame fragments, detected in all three experiments, for which the E factor is < -1 (or > 1) in all experiments. **b-c**) Replicate experiment overlap. Left (b): depleted fragments. Right (c): enriched fragments.

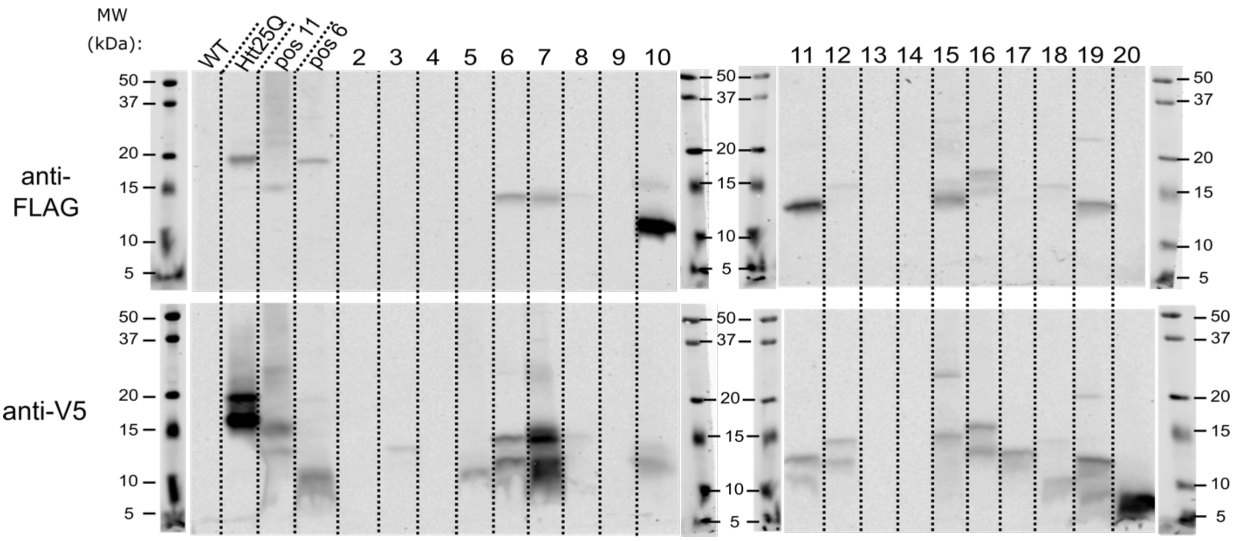

Supplementary Fig. 4. A fraction of depleted fragments are still secretable. Twenty random human fragments that were depleted after SECRiFY screening were expressed in *S. cerevisiae* with N-terminal FLAG and C-terminal V5 tag but without Sag1 anchor, and presence in medium was assessed by Western Blot. Although most fragments remained undetected, several fragments still show some level of secretability (ie bands close to the expected molecular weight detectable by FLAG and V5), suggesting a relatively low negative predictive value, as expected for a screen with focus on positive selection. DNA for fragment 1 could not be synthesized. WT: *S. cerevisiae* R1158 medium (neg. control), Htt25Q: medium from *S. cerevisiae* secreting human Htt25Q (pos. control), pos 11: enriched fragment 11 (pos. control), pos 6: enriched fragment 6 (pos. control). This experiment was performed once. MW= Molecular Weight of ladder bands (kDa). Uncropped blots are provided in the Source Data file.

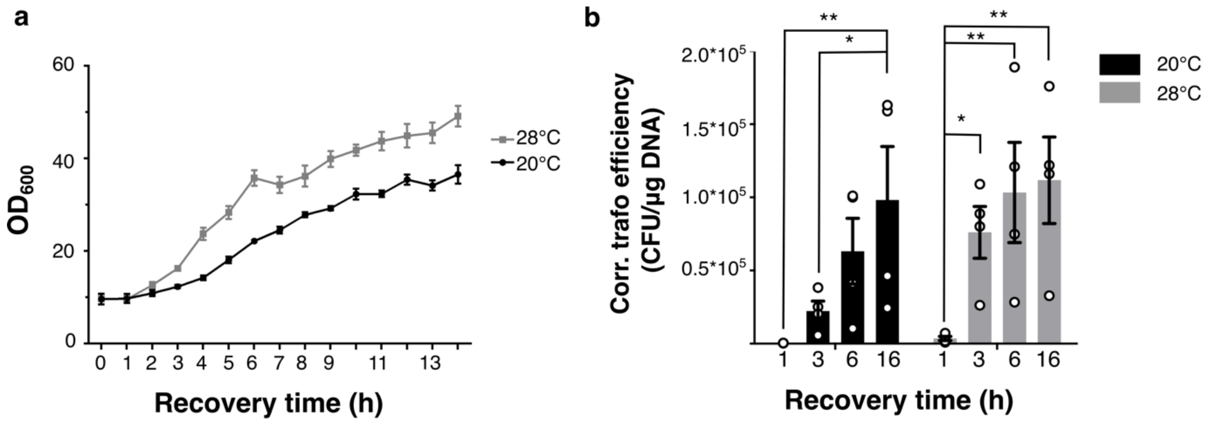

Supplementary Fig. 5. Extending recovery time of transformed *P. pastoris* cells in YPD improves transformation efficiency. a) Exponential growth of *P. pastoris* library transformants takes off after about 3h into recovery in YPD medium at 28°C after electroporation, suggesting it takes several hours for cells to recover from electroporation. Mean OD_{600} ± SEM (n= 3 biological replicates). **b**) Transformation efficiency, corrected for growth occurring during recovery, significantly increases with the length of the recovery period at both 28°C and 20°C. Two-way repeated measures ANOVA with Tukey's post-hoc test for multiple comparisons, mean corrected transformation efficiency ± SEM (n= 4 biological replicates), * p<0.05, ** p<0.01. Exact adjusted p-values: 0.0638 (20°C 1 h vs 6 h), 0.0027 (20°C 1 h vs 6 h), 0.0274 (28°C 1 h vs 3 h), 0.0023 (28°C 1 h vs 6 h), qnd 0.0010 (28°C 1 h vs 16 h). Electroporation was done with 100 ng library DNA per reaction and selection on YPD pH 8.0 + 20 µg/ml zeocin agar plates.

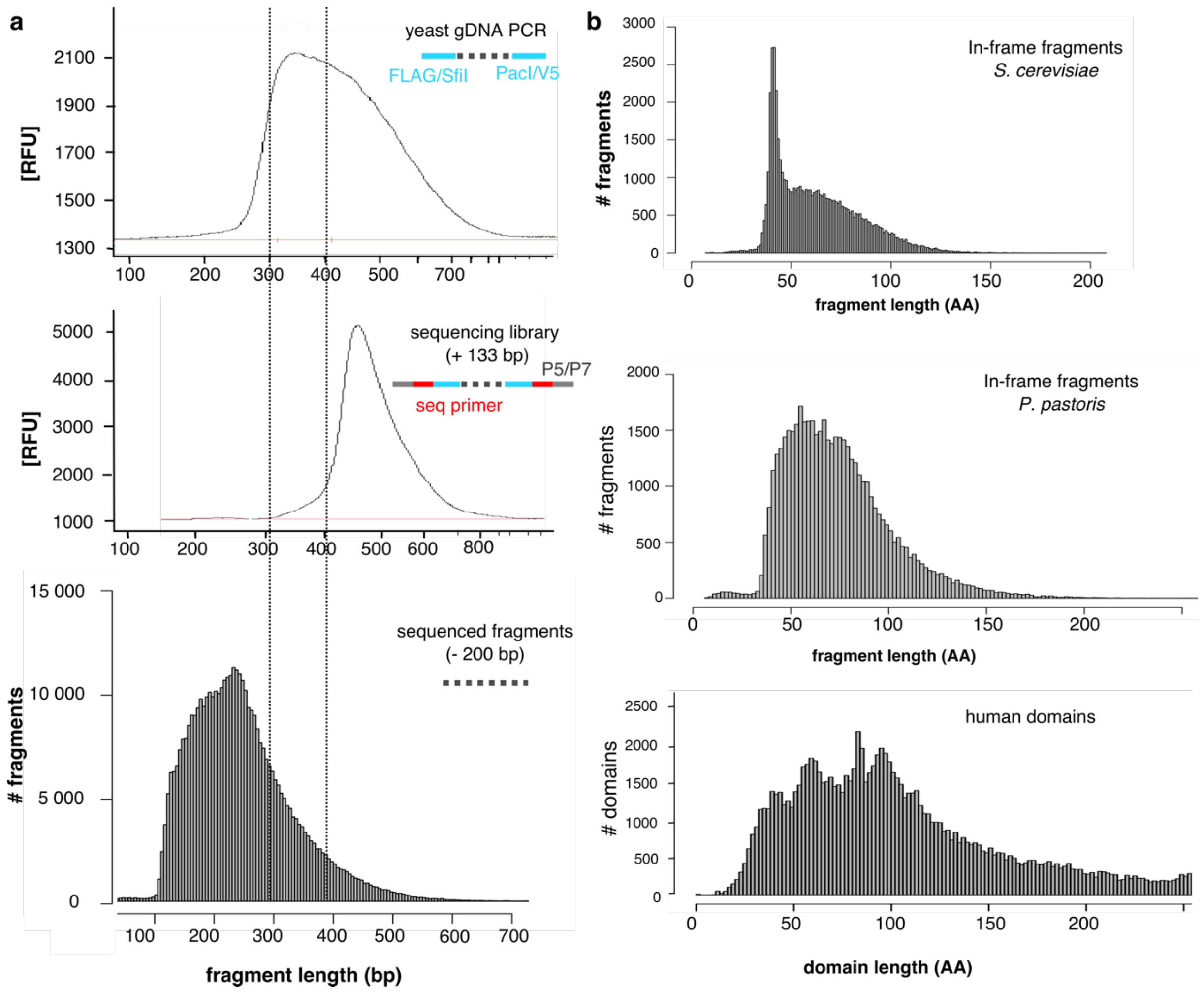

Supplementary Fig. 6. Size distributions of library cDNA fragments. a) Illumina sequencing of pools of sequences with a broad size distribution leads to a bias towards smaller fragments**.** Fragments from unsorted and sorted *P. pastoris* cells are picked up by PCR from plasmids integrated in gDNA. These fragments are between 300-500 bp in length (upper panel), including the additional 67 bp from the common priming sequences (blue). After sequencing library construction, adding the sequences necessary for paired-end sequencing (red) and flow-cell binding (gray) (+ 133 bp in total compared to the amplified fragments), total fragment length shifts accordingly, though towards a slightly narrower distribution partially depleted in the longer sequences (middle panel). The cDNA fragment distribution after sequencing and read processing (which removes 200 bp of method-related sequences) shows that most sequenced fragments are between 150-400 bp long (bottom panel, n= 423,692, *P. pastoris* experiment). Thus, we observe a bias towards smaller fragments (100-200 bp), especially during sequencing. This likely has to do with biases in bridge amplification inherent to Illumina sequencing. Such bias can likely be overcome in future experiments by using mate-pair sequencing protocols. This would also enable the analysis of libraries encoding much larger protein fragments or intact proteins **b**) Final sequenced in-frame fragment size distribution in our *S. cerevisiae* and *P. pastoris* experiments compared to that of human protein domains in Gene3D (v14.0.0, n= 104,734). The

overrepresentation of fragments smaller than 50 AA in the *S. cerevisiae* experiments (upper panel) was resolved in the *P. pastoris* experiments (middle panel) by avoiding overamplification during sequencing library prep. Nevertheless, both fragment distributions largely overlap with the size distribution of human protein domains.

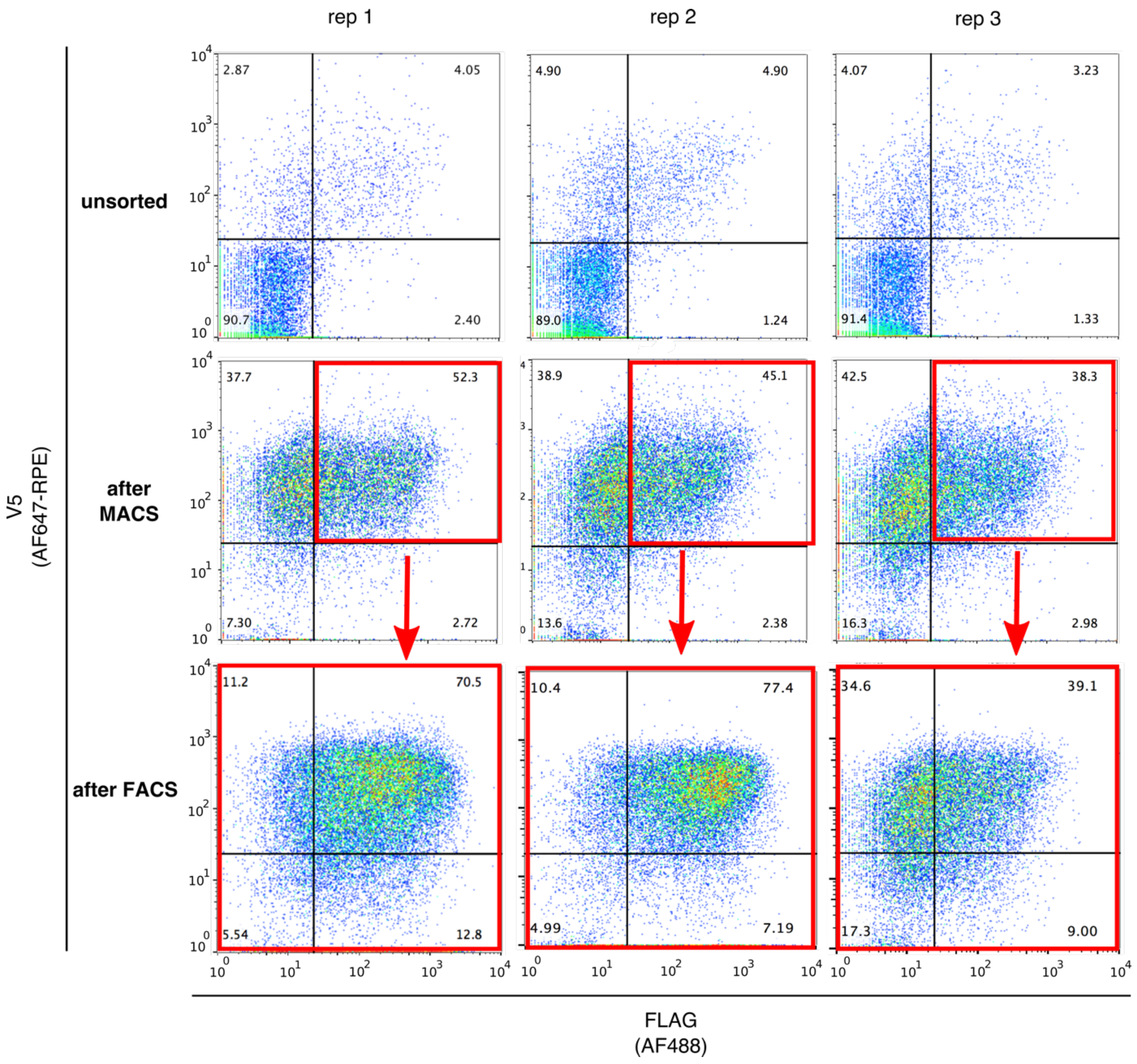

Supplementary Fig. 7. Sorting of fragment-displaying *P. pastoris* library clones. Expression of the human fragment library in *P. pastoris* was induced (upper row panels) and the pool was first enriched for $V5^+$ cells, displaying a fragment with an intact C-terminus, using MACS (middle row panels). This enriched subset was immediately sorted for $FLAG^+V5^+$ cells using FACS, representing display of fragments with both termini intact. Sorted cells were allowed to recover and were frozen for storage. The purity of these sorted cells was checked by regrowing the cells, inducing fragment expression, cell staining, and flow cytometry (lower row panels, 'after FACS'). The sorting of the library was replicated independently three times.

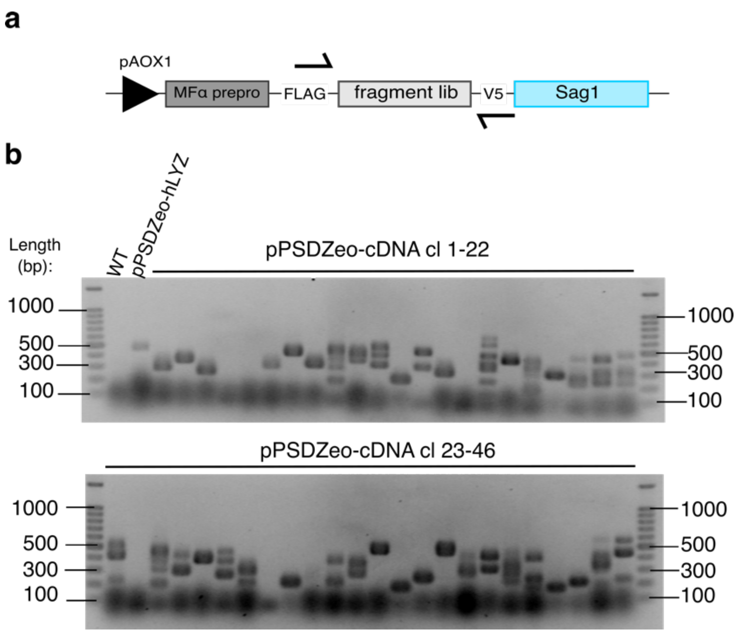

Supplementary Fig. 8. Co-integration of multiple fragments in the *P. pastoris* genome as shown by colony PCR on randomly picked library clones. a) The pPSDZeo surface display cassette in the *P. pastoris* yeast library clones. Primers for the colony PCR to amplify fragment inserts are represented by black arrows. **b)** Colony PCR of single library clones. WT= *P. pastoris* untransformed strain GS115. A strain displaying human lysozyme, marked as pPSDZeo-hLYZ, was used as a positive control and generates a band of 447 bp. Band sizes reflect the size of the fragment insert + 40 bp primer sequence. 41/46 clones contain at least one insert, 22 of those contain more than one insert. The average clone has 3 inserts. This accounts for the higher % of clones (+/- 4%, see **Supplementary Fig. 7**) that express a detectable protein fragment in the unsorted library than is the case for *S. cerevisiae* (+/- 1.7%). However, this co-integration of several fragment expression constructs will not significantly affect the reliability of determining which of these fragments are secretable: as only a tiny fraction (about 1.7%) of inserts yield a detectably surface-displayed protein fragment, the chance of co-occurrence of two such surface-displayable fragments is very low (about 1.7% of the sorted cells). This issue is further mitigated because we replicate the sorting experiment 3 times and only consider those fragments that are observed to be enriched consistently across the 3 experiments. Uncropped gels are provided in the Source Data file.

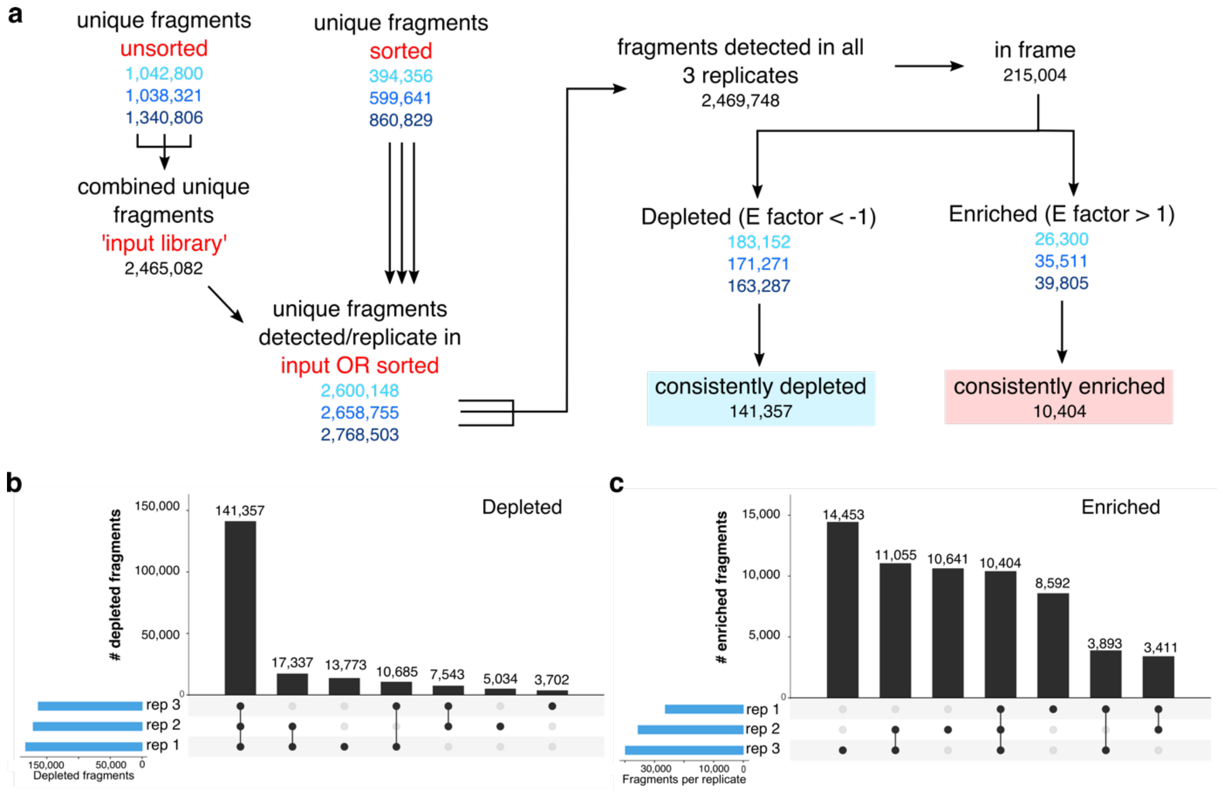

Supplementary Fig. 9. Enriched/depleted fragment classification and replicate statistics in *P. pastoris* screens. Fragment processing workflow for classification into enriched/depleted fragments, for our *P. pastoris* screens. As in Supplementary Fig. 3, the number of fragments in each of the three replicate screens is shown in different shades of blue. A fragment is considered detected in a replicate when it is detected in either the sorted sample of that experiment, and/or in the input library, which represents the total diversity of the starting yeast library before sorting to offset fragments missed due to insufficient depth of sequencing of unsorted samples. Consistently depleted (or enriched) fragments are in-frame fragments, detected in all three experiments, for which the E factor is < -1 (or > 1) in all experiments. **b-c**) Replicate experiment overlap. Left (b): depleted fragments. Right (c): enriched fragments.

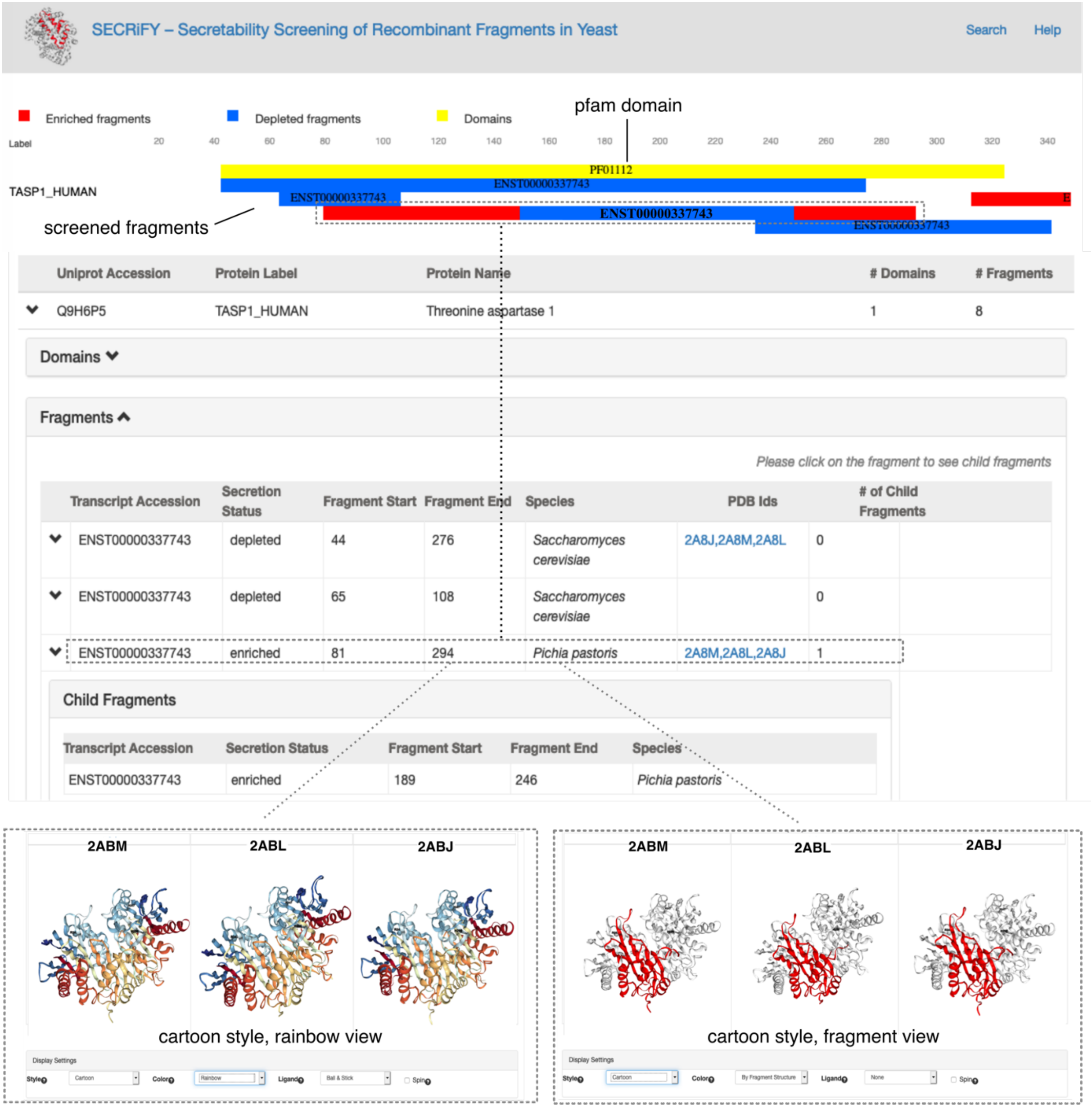

Supplementary Fig. 10. Browsing secretability data using the SECRiFY website. Users can survey which secretable or depleted fragments map to their protein of interest (here TASP1) or pfam domain of interest. The protein sequence and the SECRiFY-interrogated sequence fragments (blue for depleted, red for enriched) can be viewed using the sliding panel at the top of the page. Each fragment is labeled according to the Ensembl ID of the human transcript it mapped to. Yellow bars indicate known pfam domain regions. Pull-down panels give an overview of representative fragments (the longest fragment of a cluster of fragments with 100% overlap) and the corresponding child fragment(s) of the cluster, as well as their secretability status and the organism in which the screen was performed. Clicking on the PDB ID's to which the representative fragment mapped to enables a structural examination of the corresponding PDB files and mapped fragment in a variety of display styles (bottom insets left and right).

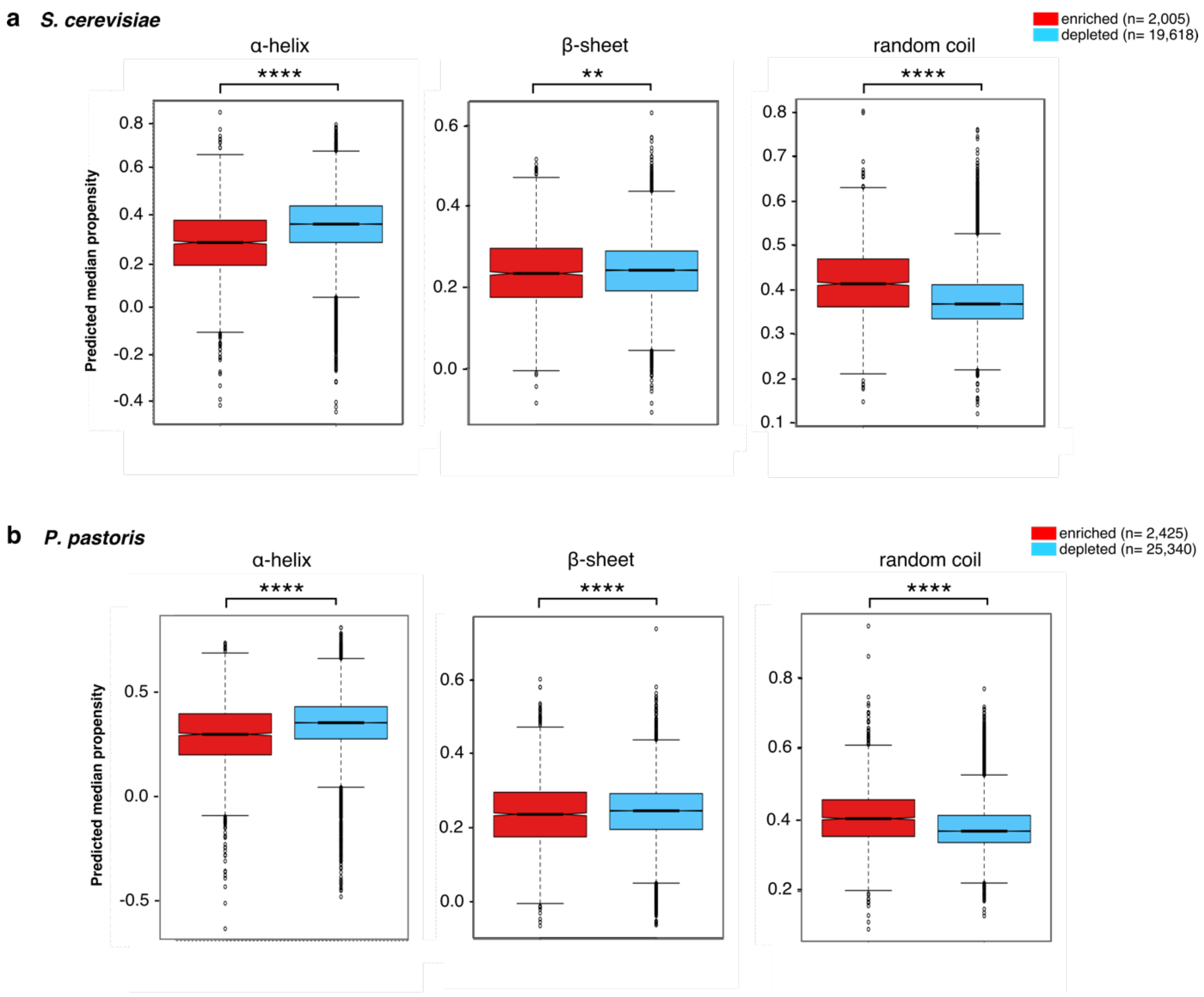

Supplementary Fig. 11. Predicted helical, sheet and random coil propensity of subsets of consolidated enriched (secretable) and depleted fragments. Box plots indicate the distribution of the median helix, sheet or coil propensity of amino acid residues, summarized per consolidated fragment. Whiskers reflect the maximum value or the respective quartile value times 1.5 the interquartile range, whichever is less. The notch displays a confidence interval based on the median plus/minus 1.57 times the interquartile range divided by the square root of the number of points. If the notches of two boxes do not overlap, this is strong evidence that their medians differ significantly. In both organisms, secretable fragments tend to have less α-helical tendencies (*S. cerevisiae:* p= $2.946*10^{-124}$*, P. pastoris:* p= $2.371*10^{-76}$*,* two-sided Mann-Whitney-Wilcoxon test), similar β-sheet propensities (*S. cerevisiae*: p= $1.262*10^{-03}$, *P. pastoris*: p= $3.360*10^{-05}$, Mann-Whitney-Wilcoxon test), and higher random coil frequencies that depleted fragments (*S. cerevisiae*: p= $1.993*10^{-127}$, *P. pastoris*: p= $1.098*10^{-85}$, two-sided Mann-Whitney-Wilcoxon test). Panel **a** was reproduced from Fig. 3 in the main text to compare with the fragments expressed in *P. pastoris* in **b**. For *S. cerevisiae*, n= 2,005 consolidated enriched and n= 19,618 consolidated depleted fragments. For *P. pastoris*, n= 2,425 consolidated enriched and n= 25,340 consolidated depleted fragments. Each consolidated fragment was detected in all 3 replicate screens.

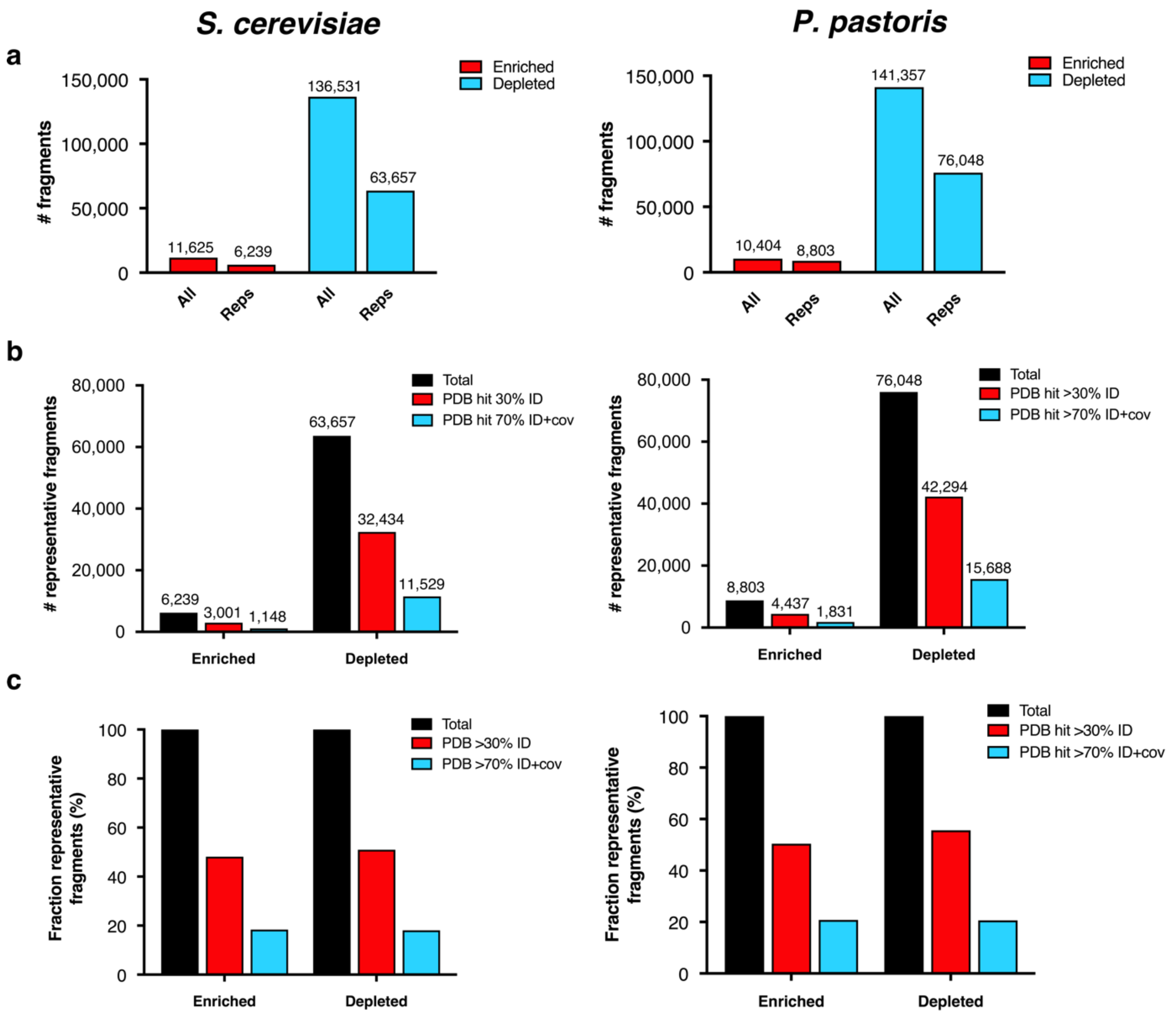

Supplementary Fig. 12. Mapping of representative fragments to PDB. a) To reduce the redundancy in the data before mapping to PDB, fragments that are 100% overlapping with longer fragments were clustered to a single representative fragment (i.e. the longest fragment). Graphs show the absolute number of fragments before and after clustering in *S. cerevisiae* (left) and *P. pastoris* (right) datasets. Depleted sets have a higher fraction of redundant fragments and were reduced to about 50% after clustering. Reps= representative fragments. **b**) Roughly 50% of the representative fragments match a sequence with a structure in PDB with ≥ 30% sequence identity, and roughly a third of those with ≥ 70% sequence identity and ≥ 70% query coverage. Data for *S. cerevisiae* (left) and *P. pastoris* (right) fragments. **c**) Same as in b but with fractional instead of absolute numbers.

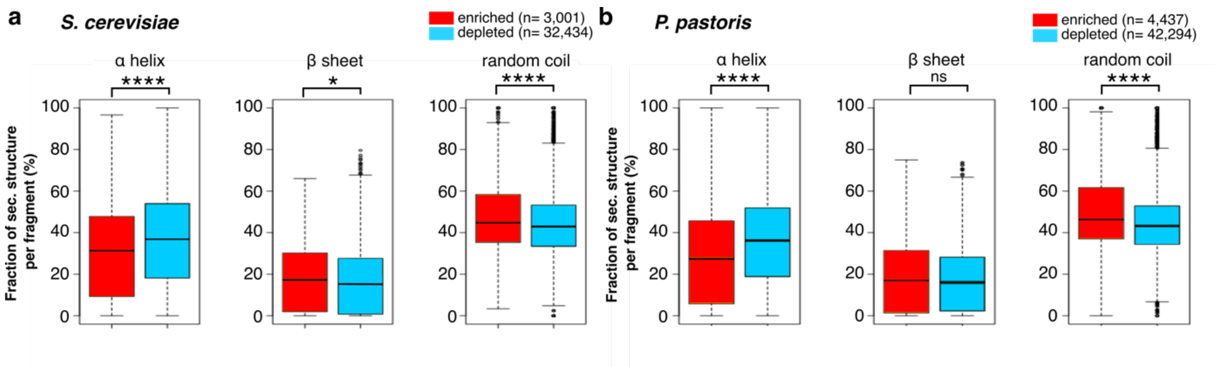

Supplementary Fig. 13. Enriched fragments on average have a lower average α-helical but higher coiled coil content. Percentage of residues in secondary structure elements in representative enriched and depleted fragments that mapped to PDB structures in both screens. Depleted fragments tend to have a higher helical propensity (*S. cerevisiae:* p= $2.35*10^{-8}$, *P. pastoris:* p= $3.12*10^{-22}$, two-sided Mann-Whitney-Wilcoxon test), whereas enriched fragments tend to have a higher coil tendency (*S. cerevisiae:* p= $1.24*10^{-4}$, *P. pastoris:* p= $2.55*10^{-14}$, two-sided Mann-Whitney-Wilcoxon test). Differences in β-sheet propensity are less pronounced (*S. cerevisiae*: p= 0.02, *P. pastoris:* p= 0.15, two-sided Mann-Whitney-Wilcoxon test). Panel **a** was reproduced from Fig. 3 in the main text to compare with the fragments expressed in *P. pastoris* in **b**. For *S. cerevisiae*, n= 3,001 enriched and n= 32,434 depleted fragments that mapped to PDB structures. For *P. pastoris*, n= 4,437 enriched and n= 42,394 depleted fragments that mapped to PDB structures. Each fragment was detected in all 3 replicate screens.

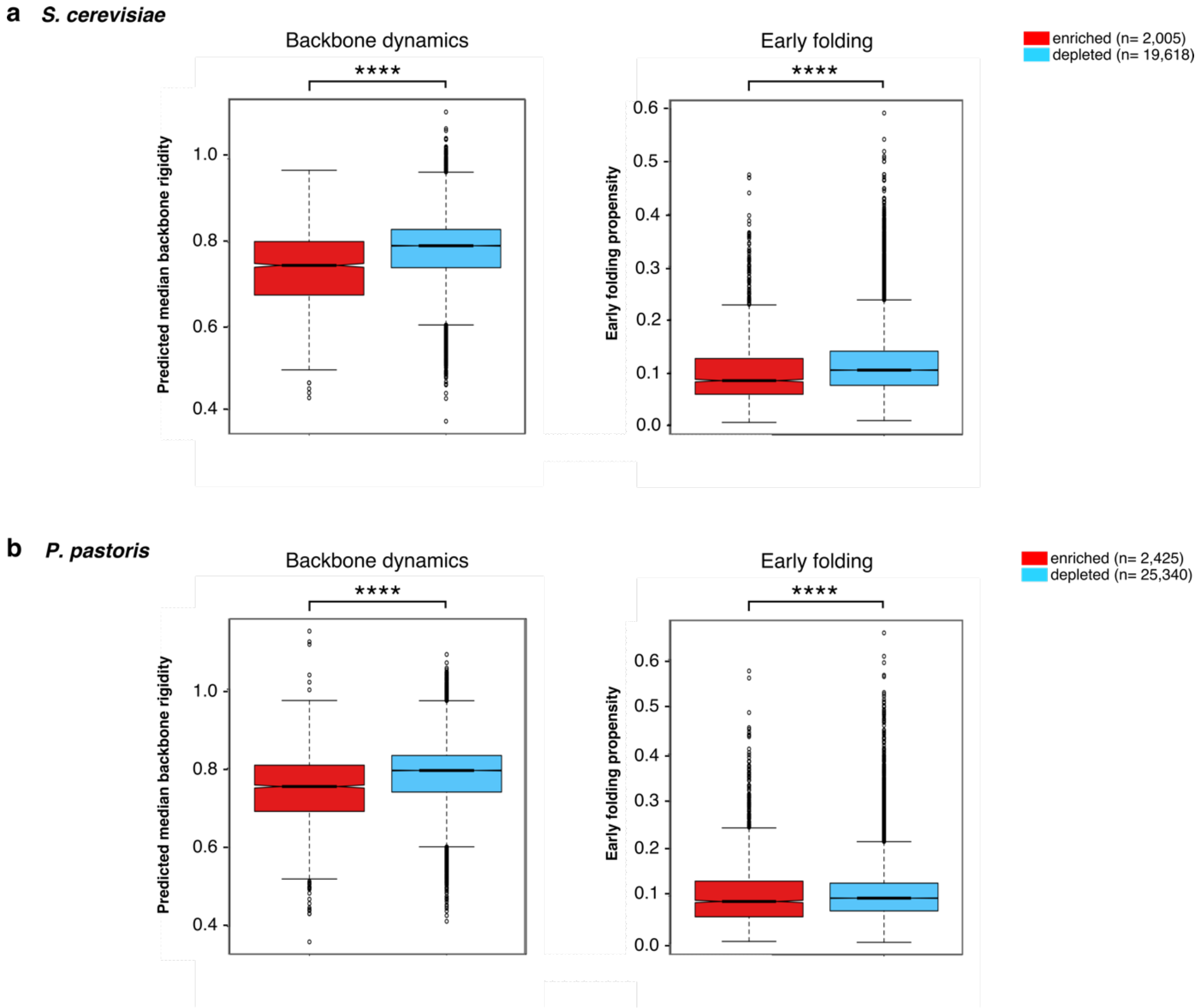

Supplementary Fig. 14. Backbone dynamics and early fold predictions. Notched box plots indicate median predicted backbone rigidity or early fold propensity of each consolidated fragment. Whiskers reflect the maximum value or the respective quartile value times 1.5 the interquartile range, whichever is less. The notch displays a confidence interval based on the median plus/minus 1.57 times the interquartile range divided by the square root of the number of points. If the notches of two boxes do not overlap, this is strong evidence that their medians differ significantly. In terms of backbone dynamics, enriched fragments score lower on the rigidity scale and seem thus to be more flexible than depleted fragments (*S. cerevisiae*: $p= 5.076*10^{-118}$, *P. pastoris*: $p= 2.498*10^{-93}$, two-sided Mann-Whitney-Wilcoxon test). The median propensity for early folding is much more similar between enriched and depleted fragments, despite significant differences in the propensity distribution (*S. cerevisiae*: $p= 2.283*10^{-52}$, *P. pastoris*: $p= 1.597*10^{-10}$, two-sided Mann-Whitney-Wilcoxon test). Panel **a** (backbone dynamics) was reproduced from Fig. 3 in the main text to compare with the fragments expressed in *P. pastoris* in **b**. For *S. cerevisiae*, n= 2,005 consolidated enriched and n= 19,618 consolidated depleted fragments. For *P. pastoris*, n= 2,425 consolidated enriched and n= 25,340 consolidated depleted fragments. Each consolidated fragment was detected in all 3 replicate screens.

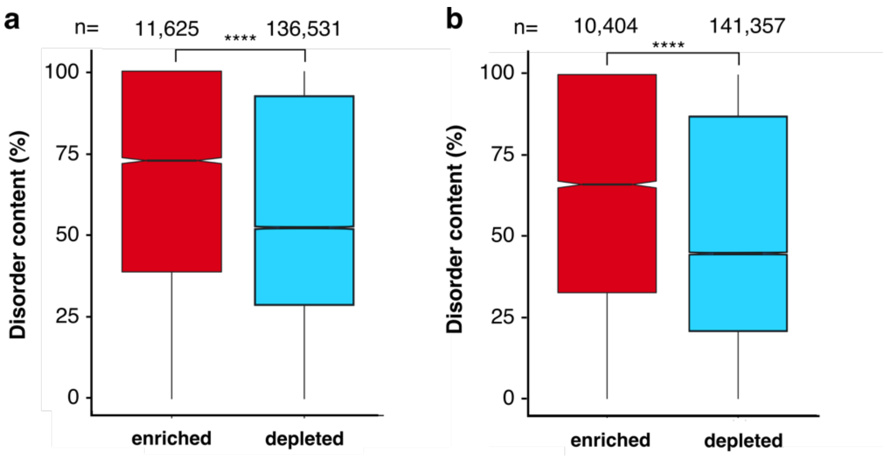

Supplementary Fig. 15. RAPID protein disorder content predictions in *S. cerevisiae* (a) and *P. pastoris* (b). Secretable fragments are predicted to have a higher average disorder content compared to depleted fragments in both organisms. Panel **a** is the same as in Fig. 3 but was reproduced here along the boxplot for the *P. pastoris* screen for comparison. Two-sided Mann-Whitney-Wilcoxon test, **** $p< 2.2*10^{-16}$. Box plots indicate the distribution of disorder content of each fragment. Whiskers reflect the maximum value or the respective quartile value times 1.5 the interquartile range, whichever is less. The notch displays a confidence interval based on the median plus/minus 1.57 times the interquartile range divided by the square root of the number of points. If the notches of two boxes do not overlap, this is strong evidence that their medians differ significantly. For **(a)** *S. cerevisiae*, n= 11,625 enriched and n= 136,531 depleted fragments. For **(b)** *P. pastoris*, n= 10,404 enriched and n= 141,357 depleted fragments. Each fragment was detected in all 3 replicate screens.

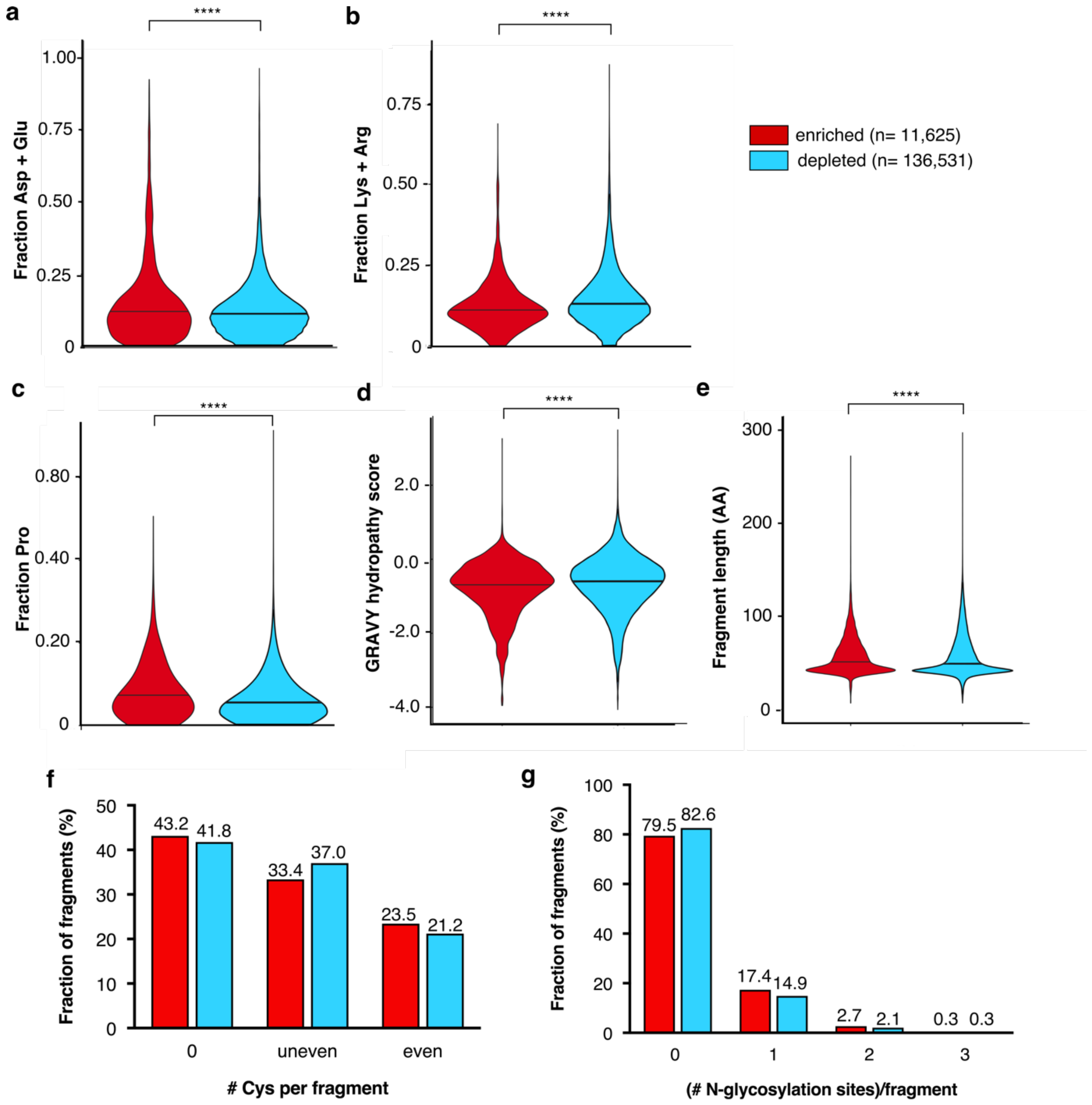

Supplementary Fig. 16. Select patterns in human fragments displayed by *S. cerevisiae*. a) Enriched fragments have a slightly larger proportion of fragments with a higher fraction of negatively charged amino acids Asp and Glu per fragment. **b**) The fraction of positively charged amino acids Lys and Arg is slightly lower in enriched fragments, which could be related to ribosome stalling on polybasic residues. **c**) The fraction of Pro is higher in enriched fragments. d) Enriched fragments tend to be less hydrophobic, as indicated with a slightly lower overall GRAVY hydropathy score. **e**) Length distributions in enriched and depleted fragments. In all violin plots (**a-e**), the black line indicates the median. Distributions were compared using the two-sided Mann-Whitney-Wilcoxon test. **** $p<2.2*10^{-16}$. **f**) Considering the oxidative environment in the ER, fragments that require paired Cys for folding could have been more prominent in enriched (secretable) fragments, but differences are only minor. **g**) Considering the important role of N-glycosylation for the folding of many secretory proteins, the number of potential N-glycosylation sites (Asn-X-Ser/Thr with X not Pro) was examined in enriched and depleted fragments, but no large differences are observed.

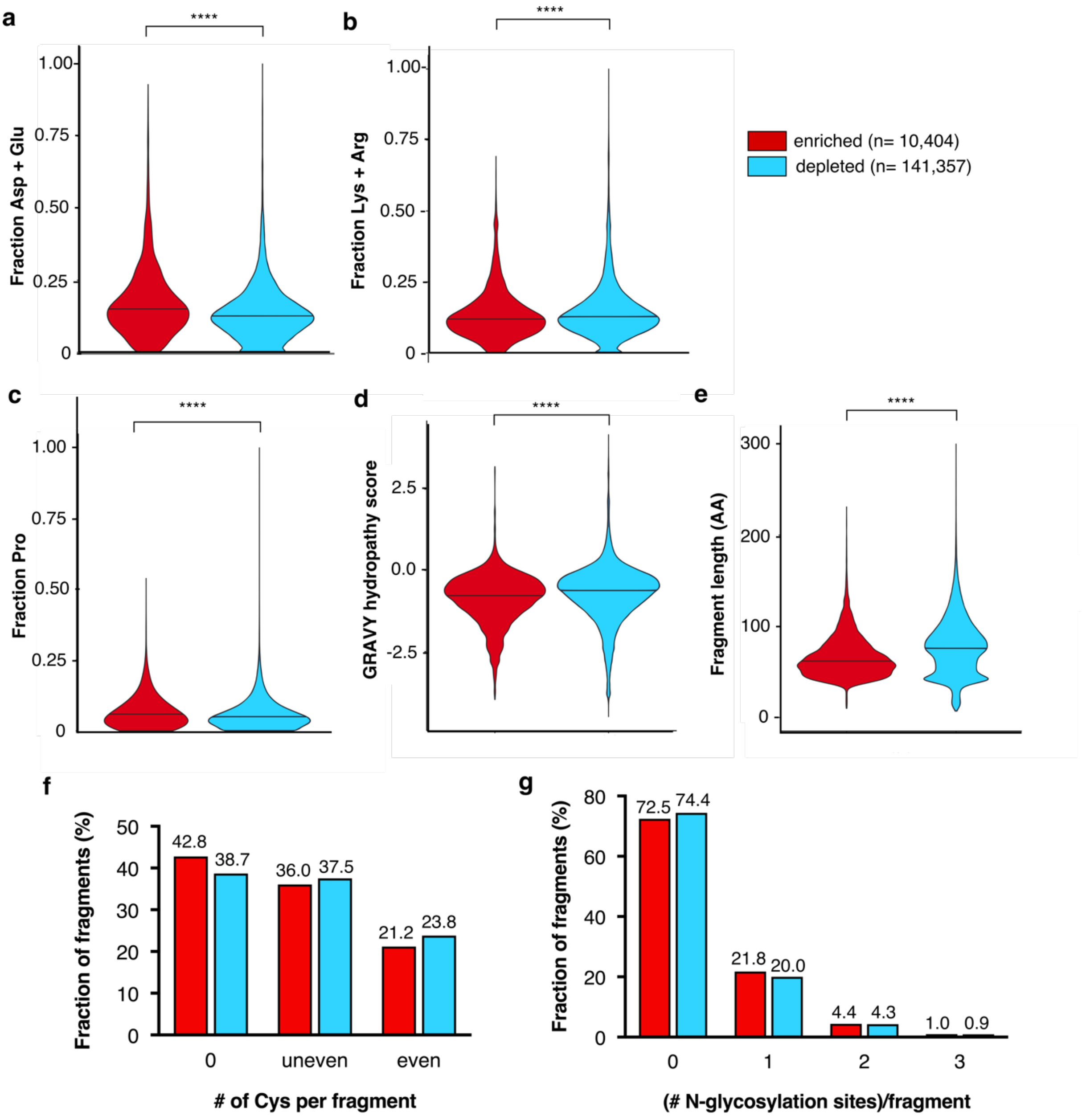

Supplementary Fig. 17. Select patterns in human fragments displayed by *P. pastoris*. We observed similar patterns in our *S. cerevisiae* screens (**Supplementary Fig. 15**): enriched fragments tend to have a slighty larger fraction of negatively charged amino acids (**a**) and Pro (**c**) (also residues that are often enriched in disordered proteins), less positively charged amino acids (**b**), less hydrophobic overall (**d**), and smaller in length (**e**). There is no clear difference in the number of Cys pairs (**f**) nor potential N-glycosylation sites (**g**). Black lines in violin plots indicate the median. Distributions were compared using the two-sided Mann-Whitney-Wilcoxon test. **** $p<2.2*10^{-16}$.

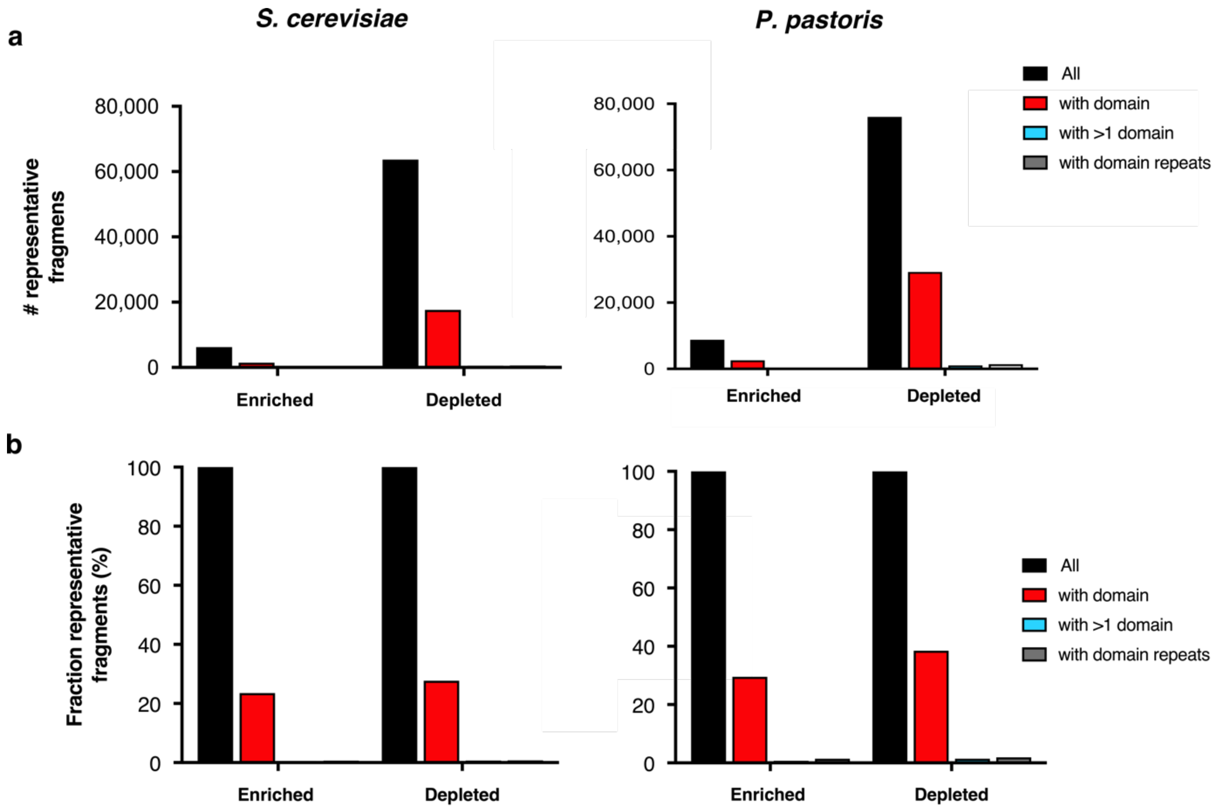

Supplementary Fig. 18. Pfam domains in enriched and depleted fragments. Number (a) and fraction (b) of representative fragments with one or more hits with Pfam domains. Left: *S. cerevisiae* screens, right: *P. pastoris* screens.

Supplementary tables

sample		# reads	Fraction of reads (%)
**total**		214,773,952	100.00
**passing filter (PF)**		199,092,922	**92.70**
**PF + barcode identified**	unsorted 1	41,979,118	21.09
	unsorted 2	41,527,810	20.86
	unsorted 3	46,443,265	23.33
	sorted 1	19,426,627	9.76
	sorted 2	18,508,105	9.30
	sorted 3	19,448,837	9.77
	**total**	187,333,764	**94.09**

sample	fraction of reads remaining after (%)	
	**Illumina sequence removal**	**FLAG/V5 trimming**
unsorted 1	95.09	95.3
unsorted 2	94.26	94.9
unsorted 3	89.39	93.8
sorted 1	92.04	94.7
sorted 2	90.43	94
sorted 3	89.82	94.2
**total**	**91.84**	**94.48**

Supplementary Table 1. *S. cerevisiae* secretability screening sequencing and trim statistics. Left: number of reads (twice the number of pairs) obtained after Illumina sequencing of 3 replicate samples. Data was summarized over four sequencing chip lanes. Of the 200 million reads passing the filter (PF), about 95% were barcode identified. Right: read statistics after trimming. In a first round, all reads matching Illumina flow cell or primer binding sequences were removed (on average about 9% of reads). Next, all FLAG+SfiI and V5+PacI sequences were trimmed off, and untrimmed or very short (< 20bp) reads were discarded, leaving roughly 95% of those sequences for mapping.

		unsorted 1	unsorted 2	unsorted 3	sorted 1	sorted 2	sorted 3
	input total reads	38,051,552	37,155,742	38,926,446	16,940,044	15,729,448	16,455,338
**R 1**	# reads	19,025,776	18,577,871	19,463,223	8,470,022	7,864,724	8,227,669
	mapped (%)	86.43	86.46	86.54	90.62	91.29	88.61
	unambiguous (%)	18.87	18.45	18.25	15.25	14.94	14.33
	ambiguous (%)	67.56	68.02	68.30	75.37	76.35	74.29
**R 2**	# reads	19,025,776	18,577,871	19,463,223	8,470,022	7,864,724	8,227,669
	mapped (%)	86.53	86.29	86.37	90.50	91.19	88.50
	unambiguous (%)	18.83	18.41	18.22	15.25	14.97	14.33
	ambiguous (%)	67.43	67.88	68.15	75.25	76.23	74.16
**total**	mated pairs (%)	84.48	84.51	84.61	88.30	88.97	86.43
	bad pairs (%)	0.86	0.83	0.84	1.45	1.36	1.33
	properly paired (%)	84.48	84.50	84.60	88.30	88.97	88.56
	singletons (%)	1.05	1.04	1.01	0.81	0.92	0.80

Supplementary Table 2. *S. cerevisiae* secretability screen mapping statistics. Reads passing the filter (PF) and barcode identified reads were mapped to the hg38 transcriptome of known protein-coding genes using BBMap. The many ambiguous read mappings (due to the presence of multiple transcripts per gene) were assigned to the single best mapped transcript for read counting. This strategy is favored over mapping to a transcriptome of only canonical transcripts as it improves the fraction of mapped reads. Downstream fragment counting was further performed using properly-paired reads only. R 1: read 1, R 2: read 2.

sample	# proper read pairs	# fragments
unsorted 1	16,072,901	991,563
unsorted 2	15,699,215	1,020,569
unsorted 3	16,466,734	1,026,175
sorted 1	7,479,200	162,329
sorted 2	6,996,856	172,142
sorted 3	7,110,914	184,576
**total**	69,825,820	//

sample	Average coverage per covered base	Bases covered (%)	Combined bases covered (%)
unsorted 1	155.18	27.40	27.55
sorted 1	356.67	3.74	
unsorted 2	155.82	26.18	26.34
sorted 2	272.65	4.39	
unsorted 3	167.5	25.29	25.46
sorted 3	259.21	4.53	

Supplementary Table 3. *S. cerevisiae* secretability screening fragment counts and transcriptome coverage statistics. Left: number of properly mapped pairs and the corresponding number of different cDNA fragments obtained per sample. Right: calculated canonical transcriptome coverage. The fraction of canonical transcriptome bases covered (‘Based covered’) concerns only those bases covered with minimum 3 properly paired reads. The combined fraction reflects the fraction of the canonical transcriptome covered per sample. Note that these are estimates, as about 5% less reads can be mapped to the canonical transcriptome, and for fragments longer than 250 bp, the gap between the paired ends is not sequenced.

Sample	median FLAG	rCV FLAG	median V5	rCV V5	unsorted count (FPTM)	sorted count (FPTM)	log2 FC
pSSD-R2 clone 1	1320.00	88.10	3353.00	104.00	60.97	1212.70	4.31
pSSD-R2 clone 2	4717.00	92.60	3586.00	79.90	0.62	30.75	5.63
pSSD-R2 clone 3	3586.00	83.30	3765.00	94.50	436.14	8614.56	4.30
pSSD-R2 clone 4	7821.00	82.30	3521.00	96.00	102.66	2432.08	4.57
pSSD-R2 clone 5	2951.00	91.10	4014.00	91.60	13.69	324.90	4.57
pSSD-R2 clone 6	1246.00	111.00	3663.00	102.00	11.82	157.77	3.74
pSSD-R2 clone 7	5184.00	90.70	2183.00	101.00	71.55	1262.17	4.14
pSSD-R2 clone 8	2573.00	90.70	3881.00	97.60	0.00	8.02	NA
pSSD-R2 clone 9	1619.00	92.10	2915.00	99.40	12.44	104.29	3.07
pSSD-R2 clone 10	385.00	92.60	3070.00	85.70	0.00	239.33	NA
pSSD-R2 clone 11	5594.00	82.00	3070.00	90.10	4.98	1218.04	7.93
pSSD-R2 clone 12	444.00	95.60	1938.00	83.20	0.00	1.34	NA
pSSD-R2 clone 13	796.00	114.00	2435.00	81.80	4.36	98.94	4.51
pSSD-R2 clone 14	607.00	77.30	1519.00	94.50	16.80	1139.16	6.08
pSSD-R2 clone 15	2604.00	80.80	2924.00	94.10	2.49	12.03	2.27
pSSD-R2 clone 16	421.00	132.00	479.00	155.00	46.04	318.22	2.79
pSSD-R2 clone 17	371.00	132.00	2751.00	86.80	NA	NA	NA
pSSD-R2 clone 18	2503.00	81.40	3033.00	92.70	1.87	13.37	2.84
pSSD-R2 clone 19	235.00	128.00	4100.00	84.00	42.93	1542.95	5.17
pSSD-R2 clone 20	4804.00	94.50	4125.00	94.50	13.07	426.52	5.03
pSSD-R2 clone 21	449.00	94.90	2802.00	88.60	115.10	1669.96	3.86
pSSD-R2 clone 22	1950.00	95.90	4789.00	89.60	97.68	3199.54	5.03
pSSD-R2 clone 23	692.00	70.40	3597.00	92.10	65.33	315.54	2.27
pSSD-R2 clone 24	1835.00	109.00	3468.00	112.00	9.33	644.45	6.11
pSSD-R2 clone 25	325.00	76.00	4051.00	119.00	138.74	5404.32	5.28
pSSD-R2 clone 26	604.00	123.00	3184.00	91.70	0.62	2625.95	12.04
pSSD-R2 clone 28	3005.00	94.20	3353.00	85.90	0.00	1.34	NA
pSSD-R2 clone 29	3941.00	78.40	3965.00	93.60	0.00	20.06	NA
pSSD-R2 clone 30	1442.00	95.40	3500.00	85.20	5.60	33.43	2.58
pSSD-R2 clone 31	305.00	71.70	3686.00	92.70	11.20	175.15	3.97
pSSD-R2 clone 32	1041.00	95.90	4292.00	96.30	0.00	1.34	NA
pSSD-R2 clone 33	9025.00	82.00	3953.00	89.10	121.94	7039.52	5.85
pSSD-R2 clone 34	2488.00	96.90	3313.00	94.70	74.04	1603.11	4.44
pSSD-R2 clone 35	1391.00	148.00	2897.00	102.00	NA	NA	NA
pSSD-R2 clone 36	2299.00	116.00	3005.00	103.00	44.80	2859.93	6.00
pSSD-R2 clone 37	367.00	123.00	3663.00	96.00	57.86	1163.23	4.33
pSSD-R2 clone 38	222.00	92.20	4214.00	86.90	54.75	119.00	1.12
pSSD-R2 clone 39	429.00	71.00	3033.00	92.30	79.01	3549.84	5.49
pSSD-R2 clone 40	551.00	105.00	3881.00	97.80	135.01	807.57	2.58
pSSD-R2 clone 41	3697.00	111.00	4398.00	92.50	2.49	22.73	3.19
pSSD-R2 clone 42	482.00	64.80	3042.00	97.30	54.13	1160.55	4.42
pSSD-R2 clone 43	4506.00	92.90	2924.00	95.90	16.18	197.88	3.61
pSSD-R2 clone 44	742.00	91.50	3204.00	91.80	149.94	3187.51	4.41
pSSD-R2 clone 45	1575.00	98.50	3343.00	92.40	0.00	1.34	NA
pSSD-R2 clone 46	2130.00	116.00	4452.00	92.60	1.24	215.26	7.43
pSSD-R2 clone 47	5344.00	106.00	3364.00	113.00	0.62	74.87	6.91

Supplementary Table 4. Randomly picked sorted clones display and fragment counts. 47 random single colonies were picked after two round sorting (replicate 1) for single-clone flow cytometric assessment of display. Values represent median FLAG or median V5 signal of the non-negative population. rCV= robust coefficient of variation. The identity of the clones was determined

by individual Sanger sequencing of the plasmid. The corresponding counts in unsorted and sorted samples as determined by sequencing are also shown. FPTM= fragments per ten million. Clone 17 and 35 were not detected in either unsorted or sorted sequenced samples.

Hit #	RefSeq or Ensembl ID	Gene Symbol	Fragment length (AA)	Expected MW FLAG-frag-V5 (kDa)
1	NM_018403	DCP1A	154	19.55
2	NM_004568,	SERPINB6	77	12.38
3	NM_001348	DAPK3	73	11.67
4	NM_023007	JMJD4	107	8.93
5	NM_005977	RNF6	58	9.36
6	NM_002503	NFKBIB	111	15.48
7	NM_006702	PNPLA6	77	11.32
8	NM_001569	IRAK1	60	9.74
9	NM_005587	MEF2A	58	9.25
10	NM_014303	PES1	45	8.36
11	NM_003870	IQGAP1	49	8.47
12	NM_001625	AK2	67	9.97
13	NM_001746	CANX	47	8.71
14	NM_015254	ANKS1A	43	7.69
15	NM_001416	EIF4A1	71	10.86
16	ENST00000553917	RALGAPA1	131	17.46
17	NM_015509	NECAP1	93	13.02
18	NM_024923	NUP120	69	10.55
19	NM_001456	FLNA	61	9.05
20	NM_022845	CBFB	118	16.84

Supplementary Table 5. Identity and expected molecular weight of the random fragments picked from sorted cells after secretion screening. Fragments were sequenced using the Sanger method, and mapped against human RefSeq transcripts for identification using BLASTn. Fragment length was calculated excluding the FLAG and V5 tags. Together, the N-terminal FLAG tag and C-terminal V5 add an additional 29 AA to the fragment (roughly 3 kDa). Note that fragment 16 maps to the C-terminus of the EIF4A1 ORF and the start of the annotated 3’ UTR, and is in a different frame than the annotated protein.

		Enriched	Depleted
replicate 1	# of fragments	20,635	147,334
	fraction of all (%)	12.12	86.55
replicate 2	# of fragments	21,480	145,806
	fraction of all (%)	12.62	85.65
replicate 3	# of fragments	20,223	147,147
	fraction of all (%)	11.88	86.44
**concordant**	# of fragments	11,625	136,531
	fraction of all (%)	**6.83**	**80.21**

Supplementary Table 6. *S. cerevisiae* enrichment and depleted fragment concordance between replicate screens. From the 170 226 common in-frame fragments, roughly 20,000 fragments, or 12.21%, are detected as enriched after sorting in each replicate, and 86.22% as depleted. About 6.83%, or 11,625 fragments, are consistently enriched in all three replicates, and 80.21% consistently depleted. The remaining 12.98% of fragments are discordant between replicates, or below the set cut-offs for enrichment/depletion classification.

sample		# reads	fraction of reads (%)
total		725,021,482	100.00
passing filter (PF)		674,935,720	**93.09**
**PF + barcode identified**	unsorted 1	180,876,078	26.80
	unsorted 2	170,163,740	25.21
	unsorted 3	156,047,813	23.12
	sorted 1	42,305,875	6.27
	sorted 2	40,564,218	6.01
	sorted 3	51,868,098	7.68
	**total**	641,825,822	**95.09**

sample	fraction of reads (%) remaining after	
	Illumina sequence trimming	FLAG/V5 trimming
unsorted 1	99.46	96.22
unsorted 2	99.49	97.09
unsorted 3	99.46	95.75
sorted 1	99.35	96.44
sorted 2	99.35	96.83
sorted 3	99.63	97.64
**total**	**99.46**	**96.66**

Supplementary Table 7. *P. pastoris* secretability screening sequencing and trimming statistics. Left: number of reads (twice the number of pairs) obtained after Illumina sequencing of 3 replicate samples. Data was summarized over four lanes. Of the roughly 675 million reads passing the Illumina quality filter (PF), about 95% were barcode identified. Right: read statistics after trimming. In a first round, all sequences matching Illumina flow cell or primer binding sequences were removed (on average less than 1% of sequences). Next, all FLAG+SfiI and V5+PacI sequences were trimmed off, and untrimmed or very short (< 20bp) reads were discarded, leaving roughly 97% of sequences for mapping.

		unsorted 1	unsorted 2	unsorted 3	sorted 1	sorted 2	sorted 3
	input total reads	173,937,472	164,365,482	148,609,312	40,534,608	39,021,504	50,455,798
**R 1**	# reads	86,968,736	82,182,741	74,304,656	20,267,304	19,510,752	25,227,899
	mapped (%)	81.02	81.16	80.31	82.67	82.82	83.74
	unambiguous (%)	21.72	21.44	20.84	20.17	20.59	20.29
	ambiguous (%)	59.30	59.72	59.48	62.49	62.23	63.45
**R 2**	# reads	86,968,736	82,182,741	74,304,656	20,267,304	19,510,752	25,227,899
	mapped (%)	80.72	80.88	80.03	82.39	82.54	83.46
	unambiguous (%)	21.63	21.36	20.75	20.11	20.52	20.22
	ambiguous (%)	59.08	59.52	59.28	62.29	62.05	63.24
**total**	mated pairs (%)	77.92	78.12	77.26	79.88	79.89	80.93
	bad pairs (%)	0.96	0.94	0.99	0.85	0.95	0.93
	properly paired (%)	77.92	78.17	77.26	79.88	79.89	80.93
	singletons (%)	1.99	1.91	1.92	1.80	1.83	1.74

Supplementary Table 8. *P. pastoris* secretability screening mapping statistics. PF, barcode-identified and trimmed reads were mapped to the human hg38 transcriptome of known protein-coding genes using BBMap. The many ambiguous read mappings, owing to the multiple transcript isoforms per gene, were assigned to a single best mapped transcript for read counting. Downstream fragment counting was further performed using properly-paired reads only.

sample	# proper pairs mapped	# fragments
unsorted 1	67,765,497	1,042,800
unsorted 2	64,238,604	1,038,321
unsorted 3	57,405,725	1,340,806
sorted 1	16,189,765	394,356
sorted 2	15,587,472	599,641
sorted 3	20,416,465	860,829
**total**	241,603,528	//

sample	Average coverage per covered base	Bases covered (%)	Combined bases covered (%)
unsorted 1	564.56	33.48	35.78
sorted 1	195.64	22.24	
unsorted 2	536.68	34.07	38.10
sorted 2	146.19	29.84	
unsorted 3	442.63	37.57	41.26
sorted 3	168.36	34.20	

Supplementary Table 9. *P. pastoris* secretability screening fragment counts and transcriptome coverage statistics. Left: number of properly mapped read pairs and the corresponding number of different cDNA fragments obtained per sample. Right: to obtain an idea of the fraction of transcriptome bases covered in our library, coverage statistics were calculated for mappings against the hg38 transcriptome of canonical transcripts of known protein coding genes. On average, every base covered by at least one read is counted about 500 times in the unsorted samples, and +/- 150 times in sorted samples. About 30% of all transcriptome bases were covered with at least 3 reads in each sample, accounting for roughly 35-40% of the transcriptome per replicate (combined sorted and unsorted fragments). Note that these are estimates, as about 5% less reads can be mapped to the canonical transcriptome, and for fragments longer than 250 bp, the gap between the paired ends is not sequenced.

		Enriched	Depleted
**replicate 1**	# of fragments	26,300	183,152
	fraction of all (%)	12.23	85.19
**replicate 2**	# of fragments	35,511	171,271
	fraction of all (%)	16.52	79.66
**replicate 3**	# of fragments	39,805	163,287
	fraction of all (%)	18.51	75.95
**concordant**	# of fragments	10,404	141,357
	fraction of all (%)	**4.84**	**65.75**

Supplementary Table 10. *P. pastoris* enrichment and depleted fragment concordance between replicate screens. From the 215 004 common in-frame fragments, between 12.2% and 18.5% are detected as enriched after sorting in each replicate, and between 76% and 85.2% as depleted. About 4.84%, or 10 404 fragments, are consistently enriched in all three replicates, and 65.75% consistently depleted. The remaining 29.41% of fragments are discordant between replicates, or below the set cut-offs for enrichment/depletion classification.

	Human		Baker's yeast	
	**Disordered**	**Not disordered**	**Disordered**	**Not disordered**
**Secretory**	831	7412	22	317
**Whole proteome**	3674	16699	924	5797

Supplementary Table 11. Disordered proteins in human and yeast secretome and proteome according to fractional disorder content. Secretory proteins here are defined as being annotated with a signal peptide in Uniprot. The number of disordered proteins is calculated as the number of proteins with a disorder content >35% as calculated with RAPID. In human, the fraction of disordered secretory proteins is significantly lower than across the whole proteome (10.1% vs 18.0%, one-sided Fisher exact test, $p<2.2*10^{-16}$). A similar trend is observed in baker's yeast (6.5% vs 13.8%, one-sided Fisher exact test, $p=2.46*10^{-5}$).

	Human		Baker's yeast	
	**Disordered**	**Not disordered**	**Disordered**	**Not disordered**
**Secretory**	2384	5859	91	248
**Whole proteome**	11390	8983	2899	3822

Supplementary Table 12. Disordered proteins in human and yeast secretome and proteome according to number of disordered amino acids. Secretory proteins here are defined as being annotated with a signal peptide in Uniprot. The number of disordered proteins here is calculated as the number of proteins with more than 50 disordered amino acids calculated with RAPID. In human, the fraction of disordered secretory proteins is significantly lower than across the whole proteome (28.9% vs 55.9%, one-sided Fisher exact test, $p<2.2*10^{-16}$). A similar trend is observed in baker's yeast (26.8% vs 43.1%, one-sided Fisher exact test, $p=9.44*10^{-10}$).

Architecture	*S. cerevisiae*					*P. pastoris*				
	# Enr.	# Depl.	Fraction Enr.	Fraction Depl.	Fold change	# Enr.	# Depl.	Fraction Enr.	Fraction Depl.	Fold Change
3 Solenoid	1	2	0.001	0.000	3.902	3	8	0.002	0.001	2.943
5-Stranded Propeller	3	9	0.004	0.001	2.601	1	12	0.001	0.001	0.654
6 Propellor	3	12	0.004	0.002	1.951	3	48	0.002	0.004	0.490
Distorted Sandwich	6	27	0.007	0.004	1.734	15	52	0.011	0.005	2.264
Beta Complex	7	32	0.009	0.005	1.707	9	41	0.007	0.004	1.723
Sandwich	63	353	0.077	0.055	1.393	137	801	0.100	0.075	1.342
Ribbon	11	65	0.013	0.010	1.321	42	181	0.031	0.017	1.821
Up-down Bundle	49	295	0.060	0.046	1.296	101	644	0.074	0.060	1.231
3-Layer(aba) Sandwich	210	1458	0.255	0.227	1.124	241	2236	0.177	0.209	0.846
7 Propellor	21	147	0.026	0.023	1.115	27	215	0.020	0.020	0.986
Roll (Alpha-Beta)	27	198	0.033	0.031	1.064	108	762	0.079	0.071	1.112
Alpha-Beta Complex	44	331	0.053	0.052	1.037	71	557	0.052	0.052	1.000
Roll (mainly Beta)	37	280	0.045	0.044	1.031	108	762	0.079	0.071	1.112
Irregular	3	23	0.004	0.004	1.018	32	65	0.023	0.006	3.863
Beta Barrel	33	259	0.040	0.040	0.994	63	405	0.046	0.038	1.221
2-Layer Sandwich	146	1180	0.177	0.184	0.965	236	1818	0.173	0.170	1.019
Alpha-Beta Barrel	24	206	0.029	0.032	0.909	28	232	0.021	0.022	0.947
Single Sheet	6	56	0.007	0.009	0.836	19	107	0.014	0.010	1.394
Alpha/alpha barrel	4	38	0.005	0.006	0.821	2	23	0.001	0.002	0.682
3-Layer(bba) Sandwich	6	57	0.007	0.009	0.821	3	82	0.002	0.008	0.287
Alpha-Beta Horseshoe	2	20	0.002	0.003	0.780	3	59	0.002	0.006	0.399
Orthogonal Bundle	83	861	0.101	0.134	0.752	151	1567	0.111	0.146	0.756
Trefoil	4	44	0.005	0.007	0.709	8	74	0.006	0.007	0.848
Alpha Horseshoe	10	333	0.012	0.052	0.234	35	530	0.026	0.049	0.518
Box	0	0	0	0	0	2	7	0.001	0.001	2.242

Supplementary Table 13. CATH domain architectures of representative enriched and depleted fragments mapping to PDB structures in *S. cerevisiae* and *P. pastoris*. While most architectures are roughly equally represented in both enriched and depleted fragments, a few architectures are found over- or underrepresented in secretable (enriched) fragments. Of those that were sampled sufficiently (detected at least in 0.5% of fragments in enriched or depleted sets), the Alpha Horseshoe architecture is substantially underrepresented in secretable fragments, while Beta Complex and Distorted Sandwiches are overrepresented in both yeasts. Fold change = fraction enriched / fraction depleted. # = number of occurrences of a particular architecture in representative enriched (Enr.) or depleted (Depl.) fragments mapping to one or multiple PDB structures. Fraction Enr. or fraction Depl. = # Enr. or Depl. / total number of all architecture occurrences in Enr. or Depl.

Dataset	# of positives	# of negatives	total
S. cerevisiae	6,194	68,062	74,256
P. pastoris	8,010	110,001	118,011

Supplementary Table 14. **Final dataset composition for (deep) machine learning training**. For both the *S. cerevisiae* and *P. pastoris* datasets, all sequences shorter than 50 amino acids were removed.

Dataset name	Species	# of positives	# of negatives	total
		(2x enriched, 1x uncertain)	(2x depleted, 1x uncertain)	
Sc_2consistent_1uncertain	*S. cerevisiae*	1,382	1,870	3,252
Pp_2consistent_1uncertain	*P. pastoris*	2,574	7,569	10,143
		(2x enriched, 1x depleted)	(2x depleted, 1x enriched)	
Sc_2consistent_1opposite	*S. cerevisiae*	4,201	5,617	9,818
Pp_2consistent_1opposite	*P. pastoris*	11,833	21,636	33,469

Supplementary Table 15. Dataset composition for fragments with just 2 consistent replicates. For both *S. cerevisiae* and *P. pastoris*, fragments were divided into subsets based on the measurements in the third replicate. All sequences shorter than 50 amino acids were removed.

Method	*S. cerevisiae*	*P. pastoris*
Polarity	0.751	0.756
Hydrophobicity	0.764	0.746
Average area buried	0.767	0.741
Buried residues	0.760	0.766
Bulkiness	0.760	0.751
Molar refraction	0.764	0.765
Recognition factors	0.760	0.742
Molecular weight	0.772	0.756
Transmembrane tendency	0.776	0.757
HPLC	0.769	0.761
Ensemble	0.781	0.772

Supplementary Table 16. Classification results using gradient boosted trees. The AUROC over a ten-fold cross-validation experiment is listed for the individual classifiers, as well as the ensemble.

Layer	Details
convolutional layer (+ ReLU)	100 filters of size 3
dropout layer	p = 0.2
max pooling layer	pool size 4
convolutional layer (+ ReLU)	100 filters of size 7
dropout layer	p = 0.2
max pooling layer	pool size 4
convolutional layer (+ ReLU)	100 filters of size 7
dropout layer	p = 0.2
variable to fixed length transformation method (if applicable)	
fully-connected layer	32 neurons
output layer (sigmoid)	1 neurons

Supplementary Table 17. Hyperparameters for the final deep learning architecture. Hyperparameters were determined by means of a grid search.

Method	# of parameters	***S. cerevisiae***	***P. pastoris***
(baseline) zero padding only	185K	0.777 (0.004)	0.767 (0.001)
global max pooling	150K	0.779 (0.002)	0.768 (0.001)
K-max pooling	162K	0.778 (0.002)	0.768 (0.001)
bidirectional GRU	330K	0.777 (0.003)	0.768 (0.001)

Supplementary Table 18. Classification results using convolutional neural networks. The average AUROC over ten ten-fold cross-validation experiments is listed, along with the standard deviation.

Dataset name	Gradient boosting	CNN
Sc_2consistent_1uncertain	0.744	0.750 (0.003)
Pp_2consistent_1uncertain	0.770	0.768 (0.001)
Sc_2consistent_1opposite	0.612	0.611 (0.003)
Pp_2consistent_1opposite	0.640	0.638 (0.002)

Supplementary Table 19. Classification results on the extra validation datasets. The AUROC is listed for the gradient boosting ensemble, as well as the average AUROC over 10 runs for the CNN with global max pooling.

Primer name	Sequence (5’->3’)
3’ AOX	GCAAATGGCATTCTGACATCC
5’ AOX	GACTGGTTCCAATTGACAAGC
A135_F	ATGTTAGGCCGAGATGGCCCAGAATTACCCTCCACGTT
A135_R	CAGTGCTTAATTAATTCCGGCTCCTATGTTGT
A136_F	CATCCAGTGGCCACTTAGGCCAAAGTCCTGA
A136_R	TCAGGACTTTGGCCTAAGTGGCCACTGGATG
A141_F	CACTAGGCCGAGATGGCC
A149_F	ATGACGATAAGGCCGAGAT
A149_R	GGAGAGGGTTAGGGATAGG
A188_F	CACTAGGCCGAGATGGCC
A188_R	GCCATCTCGGCCTAGTGACTGCTT
A196	CTTAGCATTAATTAANNNNNNN
A202_F	TCAAGGAGAAAAAACTATATCTAGATGAGATTTCCTTCAATTTT
A204_F	CTCGAGAAAAGAGAGGCC
A204_R	GACATAACTAATTACATGACTCGATTAGAATAGCAGGTACGACAAA
A207_F	GTTCGATTGCCTGTTTCT
A217_R	CGCTTCGGCCTCTCTTTTCTCGAGAGATACCCCTTCTTCTTTAG
A220_F	ATGACGATAAGGCGAGCGCCAAAAGCTCTTTTATCTCAACC
A220_R	GAGCTTTTGGCGCTCGCCTTATCGTCATCGTCTTTGTAAT
A221_F	GACATAACTAATTACATGACTC
A221_R	TCAAGGAGAAAAAACTATATC
A223_F	GATGACGATAAGGCCGAGATGGCCTAAGGACAAGGGAAAACGCAA
A223_R	GTTAGGGATAGGCTTACCATTAATTAACCCTCCTCACGTGCTATT
A237_F	AATGATACGGCGACCACCGAGATCTACACTCTTTCCCTACACGACGCTCTTCCGATC*T
A237_R_BC 1	CAAGCAGAAGACGGCATACGAGAT**TTACCGACG**GTGACTGGAGTTCAGACGTGTGCTCTTCCGATC*T
A237_R_BC 2	CAAGCAGAAGACGGCATACGAGAT**ATTGGACAC**GTGACTGGAGTTCAGACGTGTGCTCTTCCGATC*T
A237_R_BC 3	CAAGCAGAAGACGGCATACGAGAT**TCGCATGGA**GTGACTGGAGTTCAGACGTGTGCTCTTCCGATC*T
A237_R_BC 4	CAAGCAGAAGACGGCATACGAGAT**AGCGAACCT**GTGACTGGAGTTCAGACGTGTGCTCTTCCGATC*T
A237_R_BC 5	CAAGCAGAAGACGGCATACGAGAT**AGCTTCGAC**GTGACTGGAGTTCAGACGTGTGCTCTTCCGATC*T
A237_R_BC 6	CAAGCAGAAGACGGCATACGAGAT**GTCAGCCGT**GTGACTGGAGTTCAGACGTGTGCTCTTCCGATC*T
A240_F	AATGATACGGCGACCACCGA
A240_R	CAAGCAGAAGACGGCATACGA
A241_F	GCTAAAGAAGAAGGGGTAT
A241_R	AGTTTTGTGCTGGTAGTC
A247_F1	GATGACGATAAGGCCGAGAT
A247_F10	CTACTGACTGATGACGATAAGGCCGAGAT
A247_F2	TGATGACGATAAGGCCGAGAT
A247_F3	CTGATGACGATAAGGCCGAGAT
A247_F4	ACTGATGACGATAAGGCCGAGAT
A247_F5	GACTGATGACGATAAGGCCGAGAT
A247_F6	TGACTGATGACGATAAGGCCGAGAT
A247_F7	CTGACTGATGACGATAAGGCCGAGAT
A247_F8	ACTGACTGATGACGATAAGGCCGAGAT
A247_F9	TACTGACTGATGACGATAAGGCCGAGAT
A247_R1	TGGAGAGGGTTAGGGATAGG
A247_R10	GTGACATCCTGGAGAGGGTTAGGGATAGG
A247_R2	CTGGAGAGGGTTAGGGATAGG
A247_R3	CCTGGAGAGGGTTAGGGATAGG
A247_R4	TCCTGGAGAGGGTTAGGGATAGG
A247_R5	ATCCTGGAGAGGGTTAGGGATAGG
A247_R6	CATCCTGGAGAGGGTTAGGGATAGG
A247_R7	ACATCCTGGAGAGGGTTAGGGATAGG

A247_R8	GACATCCTGGAGAGGGTTAGGGATAGG
A247_R9	TGACATCCTGGAGAGGGTTAGGGATAGG
A256_F	GGATTACAAAGACGATGACGATAAGGCCGAGATGGCCGATGAGATTTCCTTCAATTTTTACTGCAGTTTTATTC GCATTAATTAATGGTAAGCCTATCCCTAACCCTCTCCTCGGTCTCGATTCTACGTAAGCGGCCGCGAATTAATT CGCCTTAGACATGACTGTT
A256_R	AACAGTCATGTCTAAGGCGAATTAATTCGCGGCCGCTTACGTAGAATCGAGACCGAGGAGAGGGTTAGGGATA GGCTTACCATTAATTAATGCGAATAAAACTGCAGTAAAAATTGAAGGAAATCTCATCGGCCATCTCGGCCTTATC GTCATCGTCTTTGTAATCC
A257_F	CGTAGAATCGAGACCGAG
A257_R	TAATCGAGTCATGTAATTAGTTATGT
A262_F	GAGGGTTAGGGATAGGCTTAC
A262_R	ACAAAGACGATGACGATAAG
B002_F	TGCACCACCAACTGCTTAGC
B002_R	GGCATGGACTGTGGTCATGAG
B003_F	GGCAATGCGGCTGCAA
B003_R	GGGTACCCACGCGAATCAC
B004_F	TGACACTGGCAAAACAATGCA
B004_R	GGTCCTTTTCACCAGCAAGCT
B005_F	CCTGGAGGAGAAGAGGAAAGAGA
B005_R	TTGAGGACCTCTGTGTATTTGTCAA
B009_F	CGGCTGTTTAACTTCGCTTC
B009_R	CACACGCCAAGAAACAGT
B012_F	ATTTGCCTTGACACCACAACA
B012_R	CCTTAACTGGACCTGTACTGTGA
B013_F	AGTCACAGTGAGAAGGCGAC
B013_R	CAATTCGGCAAAACTCTGCTG
B015_F	TACCAAGTGTCGCCTTTTAACC
B015_R	GCTCTTGTCTGCATAACAGAACT

Supplementary Table 20. Primers used in this study. *= phosphorothioate. Bold underlined=barcode

Supplementary References

1. Mattanovich, D. et al. Genome, secretome and glucose transport highlight unique features of the protein production host *Pichia pastoris*. *Microb. Cell Factories* **8,** 29 (2009).
2. Ahmad, M., Hirz, M., Pichler, H. & Schwab, H. Protein expression in *Pichia pastoris*: recent achievements and perspectives for heterologous protein production. *Appl. Microbiol. Biotechnol*. **12**, 5301-5317 (2014).
3. Djebali, S. et al. Landscape of transcription in human cells. *Nature* **489**, 101-108 (2012)
4. Lyamichev, V., Brow, M.A., & Dahlberg, J. E. Structure-specific endonucleolytic cleavage of nucleic acids by eubacterial DNA polymerases. *Science* **260**, 778–783 (1993).
5. Xu, Y. et al. Biochemical and mutational studies of the 5’-3’ exonuclease of DNA polymerase I of *Escherichia coli*. *J. Mol. Biol*. 268, 284–302 (1997).
6. Davis, C., Barvish, Z. & Gitelman, I. A method for the construction of equalized directional cDNA libraries from hydrolyzed total RNA. *BMC Genomics* **8**, 363 (2007).
7. Howland, S. W., Poh, C.-M. & Rénia, L. Directional, seamless, and restriction enzyme free construction of random-primed complementary DNA libraries using phosphorothioate-modified primers. *Anal. Biochem*. **416**, 141–143 (2011).
