# Supplementary material for: Massively parallel interrogation of protein fragment secretability using SECRiFY reveals features influencing secretory system transit
Source: Nat Commun. 2021 Nov 5;12:6414. doi: 10.1038/s41467-021-26720-y (PMC8571348; doi:10.1038/s41467-021-26720-y)
Supplement: Supplementary file 2 — Reporting Summary [file 41467_2021_26720_MOESM2_ESM.pdf]

nature research

Corresponding author(s): Boone, Morgane and Callewaert, Nico

Last updated by author(s): Oct 6, 2021

Reporting Summary

Nature Research wishes to improve the reproducibility of the work that we publish. This form provides structure for consistency and transparency in reporting. For further information on Nature Research policies, see our Editorial Policies and the Editorial Policy Checklist.

Statistics

For all statistical analyses, confirm that the following items are present in the figure legend, table legend, main text, or Methods section.

n/a	Confirmed	
☐	☒	The exact sample size (*n*) for each experimental group/condition, given as a discrete number and unit of measurement
☐	☒	A statement on whether measurements were taken from distinct samples or whether the same sample was measured repeatedly
☐	☒	The statistical test(s) used AND whether they are one- or two-sided<br>*Only common tests should be described solely by name; describe more complex techniques in the Methods section.*
☒	☐	A description of all covariates tested
☐	☒	A description of any assumptions or corrections, such as tests of normality and adjustment for multiple comparisons
☐	☒	A full description of the statistical parameters including central tendency (e.g. means) or other basic estimates (e.g. regression coefficient) AND variation (e.g. standard deviation) or associated estimates of uncertainty (e.g. confidence intervals)
☒	☐	For null hypothesis testing, the test statistic (e.g. *F*, *t*, *r*) with confidence intervals, effect sizes, degrees of freedom and *P* value noted<br>*Give P values as exact values whenever suitable.*
☒	☐	For Bayesian analysis, information on the choice of priors and Markov chain Monte Carlo settings
☒	☐	For hierarchical and complex designs, identification of the appropriate level for tests and full reporting of outcomes
☐	☒	Estimates of effect sizes (e.g. Cohen's *d*, Pearson's *r*), indicating how they were calculated

Our web collection on statistics for biologists contains articles on many of the points above.

Software and code

Policy information about availability of computer code

Data collection

sequencing data was collected on an Illumina NextSeq 500 using the NextSeq System Suite 2.0.2

Data analysis

Sequencing data analysis: raw reads were first trimmed with Trim Galore! version 0.4.1 (www.bioinformatics.babraham.ac.uk/projects/trim_galore) to remove Illumina adapter sequences. Next, FLAG/V5 and frameshifting sequences were trimmed off with Cutadapt version 1.10 (ref. 10), discarding all untrimmed pairs to only keep correctly cloned cDNA fragments. Quality control of raw and processed fastq files was performed using FastQC version 0.11.3 (www.bioinformatics.babraham.ac.uk/projects/fastqc). Processed reads were mapped to the human transcriptome of known protein-coding genes as downloaded from Ensembl's BioMart11 using BBMap v35.40 (sourceforge.net/projects/bbmap). Count tables were built and analyzed from the properly paired mapped reads using SAMtools12 v1.2 and v1.3, BEDtools13 v2.24.0 and v2.25.0, EMBOSS14 v6.6.0, R project 3.3.0 (www.R-project.org) and the R packages plyr (v1.8.6), ggplot2 (v3.3.4), alakazam (v1.1.0), stringr (v1.4.0), and UpSetR (v1.4.0). A summary of the most important scripts can be found on Figshare (figshare.com/s/5dba6b512fa74ef68a40).
Structural bioinformatics: Secondary structure (a-helix, b-sheet and random coil) and early folding propensities were predicted using EFoldMine. Backbone dynamics of sequenced fragments were predicted using Dynamine. For PDB mapping, protein fragment sequences were first clustered into representative fragments using the CD-HIT package. The representative fragments were blasted against the PDB database using standalone blast (ncbi-blast-2.6.0+). The percentage of secondary structural elements for each fragment with a PDB hit was calculated from its corresponding DSSP coordinates. Domain architectures (Pfam and Gene3D) were retrieved using InterProScan22 (v 5.24-63.0). The code to generate the Pfam hits and PBD mapping can be accessed via Github (https://github.com/Pathmanaban/SECRiFY_PDB_processing, DOI: 10.5281/zenodo.5542734).
Machine Learning: The code for training, evaluating and visualizing a convolutional neural network for secretability prediction can be found at Github (https://github.com/jasperzuallaert/SecrifyDL, DOI: 10.5281/zenodo.5541041), as can the code for the gradient boosted decision tree modeling (https://github.com/RobbinBouwmeester/SECRiFY_xgb, DOI: 10.5281/zenodo.5541418).
GraphPad Prism v7 and v9 were used for visualization and statistics of qPCR bar graphs, P. pastoris growth experiments, and S. cerevisae correlation between flow cytometry median fluorescence intensity and enrichment factors.

FlowJo v10.1 was used for flow cytometry data analysis.

For manuscripts utilizing custom algorithms or software that are central to the research but not yet described in published literature, software must be made available to editors and reviewers. We strongly encourage code deposition in a community repository (e.g. GitHub). See the Nature Research guidelines for submitting code & software for further information.

Data

Policy information about availability of data

All manuscripts must include a data availability statement. This statement should provide the following information, where applicable:

- Accession codes, unique identifiers, or web links for publicly available datasets
- A list of figures that have associated raw data
- A description of any restrictions on data availability

Source data are provided with this paper. Unprocessed fastq files of both screens have been deposited in the Sequence Read Archive (SRA) under BioProject accession code PRJNA357179 (https://www.ncbi.nlm.nih.gov/sra/?term=PRJNA357179). Lists of all detected, enriched or depleted fragments are available on Figshare (figshare.com/s/82bb61370d7024f6fb09 for S. cerevisiae screens and figshare.com/s/cace104b0ffc5a57811f for P. pastoris screens), as are the lists of Pfam hits for all representative fragments (figshare.com/s/052370ec40154c09fb68), and CATH/Gene3D hits for those fragments mapping to PDB structures (figshare.com/s/5a8ca88d27168243c9fe). The data has been integrated in a web interface, available at http://iomics.ugent.be/secrify/search, for easy browsing by biologists interested in secretability of fragments of particular proteins of interest (Supplementary Fig. 10). These fragments are visually mapped to the PDB model of the protein's structure, where such structure is available.

Field-specific reporting

Please select the one below that is the best fit for your research. If you are not sure, read the appropriate sections before making your selection.

☒ Life sciences ☐ Behavioural & social sciences ☐ Ecological, evolutionary & environmental sciences

For a reference copy of the document with all sections, see nature.com/documents/nr-reporting-summary-flat.pdf

Life sciences study design

All studies must disclose on these points even when the disclosure is negative.

Sample size	No sample size calculation was performed, sample size was determined empirically. For experiments presenting mean or media data, a minimum of 3 biological replicates was performed unless otherwise stated, as is standard practice in the field.
Data exclusions	No data was excluded.
Replication	Each screening sort was replicated independently 3 times, starting from the same unsorted yeast library. The qPCR normalization efficiency determination experiments were performed with 2-3 biological replicates (Fig 1f) and 9 biological replicates (Fig 1g). For Pichia growth experiments, OD600 was measured using 3 biological replicates per condition (Supp Fig 5a) and cloning efficiency using 4 biological replicates per condition (Supp Fig 5b).
Randomization	No randomization was performed, as standard practice in the field. Libraries were pooled for sequencing.
Blinding	No blinding was performed for sequencing or cellular studies, as is standard practice in the field.

Reporting for specific materials, systems and methods

We require information from authors about some types of materials, experimental systems and methods used in many studies. Here, indicate whether each material, system or method listed is relevant to your study. If you are not sure if a list item applies to your research, read the appropriate section before selecting a response.

Materials & experimental systems

n/a	Involved in the study	
☐	☒	Antibodies
☐	☒	Eukaryotic cell lines
☒	☐	Palaeontology and archaeology
☒	☐	Animals and other organisms
☒	☐	Human research participants
☒	☐	Clinical data
☒	☐	Dual use research of concern

Methods

n/a	Involved in the study	
☒	☐	ChIP-seq
☐	☒	Flow cytometry
☒	☐	MRI-based neuroimaging

Antibodies

Antibodies used	Western Blot: polyclonal rabbit anti-FLAG antibody (1/2000, Sigma, F7425); anti-rabbit IgG-Dylight800 antibody (1/15000, Thermo

	Scientific, #35571); mouse anti-V5 monoclonal antibody (1/3000, AbD Serotec, #MCA1360); anti-mouse IgG-Dylight8000 (1/15000, Thermo Scientific, #35521).<br>Flow cytometry: mouse anti-V5 monoclonal (1/500, AbD Serotec MCA2892), rabbit polyclonal anti-FLAG (1/200, Sigma-Aldrich F7425), goat anti-mouse AF647-RPE (1/250, Life Technologies A20990), goat anti-rabbit AF488 (1/500, Life Technologies A11008)
Validation	polyclonal rabbit anti-FLAG antibody (Sigma, F7425): validated using whole extract of human HEK293T overexpressing N-terminal FLAG-tagged fusion protein by the manufacturer (https://www.sigmaaldrich.com/US/en/product/sigma/f7425),as well as in-house using positive controls (FLAG-V5-Sag1 surface display FLAG only control by flow cytometry, Supp Fig 2) and E. coli lysate with FLAG-tagged protein (Fig 2d).<br>mouse anti-V5 monoclonal antibody (AbD Serotec, #MCA1360): validated by manufacturer using a V5-tagged recombinant protein (https://www.bio-rad-antibodies.com/monoclonal/viral-v5-tag-antibody-sv5-pk1-mca1360.html?f=purified), and in-house using positive controls (FLAG-V5-Sag1 surface display V5 only control by flow cytometry, Supp Fig 2) and E. coli lysate with V5-tagged protein for Western Blot (Fig 2d).<br>mouse anti-V5 monoclonal antibody(AbD Serotec MCA2892): validated by manufacturer using a V5-tagged recombinant protein (https://www.bio-rad-antibodies.com/monoclonal/viral-v5-tag-antibody-sv5-pk2-mca2892.html?f=purified), and in-house using positive controls (FLAG-V5-Sag1 surface display V5 only control by flow cytometry, Supp Fig 2) and E. coli lysate with V5-tagged protein for Western Blot (Fig 2d).

Eukaryotic cell lines

Policy information about cell lines

Cell line source(s)	VIB IRC cell bank (HEK293T, HepG2, MCF7-AZ, and SK-N-SH) , Coriell Institute (GM12787)
Authentication	no cell line authentication was performed.
Mycoplasma contamination	all cell lines were routinely tested for mycoplasma contamination (none found)
Commonly misidentified lines<br>(See ICLAC register)	no commonly misidentified lines were used.

Flow Cytometry

Plots

Confirm that:

- [x] The axis labels state the marker and fluorochrome used (e.g. CD4-FITC).
- [x] The axis scales are clearly visible. Include numbers along axes only for bottom left plot of group (a 'group' is an analysis of identical markers).
- [x] All plots are contour plots with outliers or pseudocolor plots.
- [x] A numerical value for number of cells or percentage (with statistics) is provided.

Methodology

Sample preparation	See section 'S. cerevisiae cell sorting' and 'P. pastoris cell soring' in Materials and Methods for a detailed description of sample preparation.
Instrument	MoFlo Legacy sorter (Beckman Coulter)
Software	FlowJo v10.1
Cell population abundance	See Supplementary Figures 1 and 7.
Gating strategy	Cells were gated for a uniform SSC vs FSC single-cell population, and fluorescence quadrant gates were chosen as such that, after compensation, max. 5% of cells of unstained and single stained controls appeared above the background.

- [x] Tick this box to confirm that a figure exemplifying the gating strategy is provided in the Supplementary Information.
